# Disentangling Adsorption and Absorption in Microporous Polymers

**DOI:** 10.1002/smtd.202500845

**Published:** 2025-08-07

**Authors:** Máté Füredi, Andrei Kolesnikov, Anqi Wang, Klara Burdova, Natan Abelian, Sunshine Iguodala, Bálint Fodor, Gennady Y. Gor, Qilei Song, Stefan Guldin

**Affiliations:** ^1^ Department of Chemical Engineering University College London London WC1E 7JE UK; ^2^ Semilab Co. Ltd Budapest 1117 Hungary; ^3^ Otto H. York Department of Chemical and Materials Engineering New Jersey Institute of Technology Newark NJ 07102 USA; ^4^ Department of Chemical Engineering Imperial College London London SW7 2AZ UK; ^5^ Department of Life Science Engineering Technical University of Munich 85354 Freising Germany; ^6^ TUMCREATE Singapore 138602 Singapore

**Keywords:** adsorption, ellipsometric porosimetry, microporous materials, polymers, thin films

## Abstract

Polymers of intrinsic microporosity (PIMs) are a unique class of soft materials, which, unlike hard materials such as zeolites or carbons, are capable of both vapor adsorption (nanopore filling) and absorption (polymer plasticization/swelling). While adsorption is governed by pore structure and surface functionalization, adsorption depends on the chemical composition of the matrix material. Since both adsorption and absorption increase sorbent mass (vapor uptake), gravimetric and volumetric characterization methods exhibit severe limitations in isotherm interpretation. Thus, distinguishing between vapor adsorption and absorption remains a key challenge for understanding sub‐nanometer‐scale processes, which play a crucial role in many emerging applications of PIMs, including gas separation, water purification, organic solvent nanofiltration, and electrochemical energy storage/conversion. Herein, an alternative approach is presented based on in situ ellipsometric read out and concurrent optical modeling of adsorption and absorption. Ellipsometry is highly sensitive to changes in thickness and optical density of the thin film sorbents, enabling the acquisition of both adsorption and absorption isotherms. This study showcases four PIM sorbents with varied material chemistry, responding to various vapors. Their ad‐/absorption contributions are studied and disentangled experimentally, with nanopore confinement and swelling models based on classical physisorption and polymer Flory‐like theory.

## Graphical abstract

The dual gas adsorption‐absorption behavior of microporous polymers with varied material chemistry is disentangled in this work via an in situ optical approach and further elucidated via models from classical physisorption and polymer lattice theory.

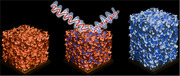

## 1 Introduction

Microporous materials defined by pores less than 2 nm in width,^[^ ^1^, ^2^, ^3^, ^4^, ^5^ ^]^ facilitate exceptionally high surface area,^[^ ^6^, ^7^, ^8^ ^]^ making them attractive for various applications including energy storage,^[^ ^9^ ^]^ environmental sensors,^[^ ^10^ ^]^ gas separation,^[^ ^11^, ^12^ ^]^ thin film optics,^[^ ^13^ ^]^ and membrane technologies.^[^ ^14^, ^15^, ^16^ ^]^ Among these, porous organic polymers are particularly sought‐after due to their highly tunable structures in the Ångström‐to‐nanometer‐scale, which can be tailored for a wide range of membrane applications.^[^ ^16^, ^17^, ^18^ ^]^

Polymers of intrinsic microporosity (PIMs)^[^ ^19^, ^20^ ^]^ are one class of such microporous materials with a key advantage of solution processability,^[^ ^21^ ^]^ enabling simple evaporation‐induced deposition of large‐area thin films or free‐standing membranes with tunable thickness from nanometers to millimeters. PIMs feature a rigid and contorted macromolecular structure that prevents efficient chain packing in the solid state, resulting in a highly interconnected microporous network, with typical pore width distributions of 0.3–1.0 nm.^[^ ^20^, ^22^ ^]^ Their modular synthesis enables the introduction of diverse functionalities, allowing material properties such as hydrophobicity to be tailored for specific applications. The archetypal PIM‐1 is relatively hydrophobic and well suited for gas separation and non‐aqueous applications, such as organic solvent nanofiltration and batteries.^[^ ^23^, ^24^, ^25^, ^26^ ^]^ Fluorine‐rich PIMs exhibit improved hydrophobicity and maintain high gas separation selectivity in humid environments. PIMs with hydrophilic or charged functional groups, on the contrary, have been developed as ion‐conducting thin‐film membranes for aqueous redox flow batteries and ultra‐thin ion‐selective protection layer on zinc electrode in zinc‐ion batteries.^[^ ^27^, ^28^, ^29^, ^30^ ^]^

Gas or vapor sorption techniques allow for measurements of various material properties relevant to PIMs, such as diffusion constants, surface area, or pore size distributions. In general, gravimetric/volumetric sorption techniques are routinely used for micropore assessment, with common adsorptive including nitrogen, argon, or carbon dioxide.^[^ ^31^ ^]^ However, these techniques are primarily designed for bulk samples and show reduced accuracy when applied to sub‐micrometer thin films.^[^ ^32^, ^33^ ^]^ One major reason is due to the very low absolute mass of the ultrathin PIM adsorbents, which amplifies errors in volumetric/gravimetric analysis. Furthermore, previous works reported significant plasticization behavior exhibited by glassy polymers such as PIMs (e.g. PIM‐1) when exposed to organic vapors, or even high‐pressure CO_2_.^[^ ^12^, ^34^, ^35^, ^36^ ^]^ Exposure to these fluids preferential to the PIM causes it to simultaneously undergo a double‐effect of adsorption via pore‐filling and absorption via swelling/plasticization, leading to a variety of changes in its mechanical properties, volume, and permeability. Importantly, the absorption effect entangles the volumetric/gravimetric vapor uptake isotherms,^[^ ^37^, ^38^ ^]^ which do not follow the IUPAC type I shape^[^ ^2^ ^]^ for most PIMs, as opposed to expectations based on conventional (hard) microporous materials such as zeolites or carbide‐derived carbons. These limitations necessitate new experimental and model‐based approaches for PIM isotherm acquisition and interpretation, especially when polymers are fabricated into submicron thin films.

In this context, optical techniques conceptually offer novel pathways to disentangle adsorption and absorption. Adsorption, i.e. the microscopic adhesion of gas molecules to the surface of nanoscale pores, and macroscopic absorption, inducing the swelling and plasticization of the polymer matrix, result in opposing effects on optical density. The principal attributes describing these competing processes, i.e. the effective refractive index (*n*) and thickness (*h*) of the films, can both be readily measured by spectroscopic ellipsometric (SE) read out under controlled atmospheric conditions. As a vapor adsorptive enters and confines within open pores, *n* across the film increases, due to phase change from what would usually be ‘air pockets’ (*n*_vacuum_ = 1.00) to that of condensed phase adsorptive (e.g. *n_water_* ≈1.33, *n_methanol_* ≈1.33, *n_ethanol_* ≈1.36, all taken at *λ =* 632 nm). On the other hand, during absorption process, swelling causes *n* to decrease as the vapor penetrates the polymer matrix (*n*_polymer_≈1.5 at *λ* *=* 632 nm), reducing its optical density. This effect is also complemented by a significant increase in film thickness, which is simultaneously monitored and acquired through SE data analysis.^[^ ^39^, ^40^ ^]^

There has been an increasing number of instances in which porous structures have been characterized using ex situ or in situ SE, providing accurate measurements of the film's optical constants (refractive index and extinction coefficient dispersions) and its thickness.^[^ ^41^, ^42^ ^]^ In essence, ellipsometry measures the change in the polarization state of light upon reflection (or transmission) from the thin film sample at a set spectral wavelength (*λ*) range.^[^ ^43^ ^]^ The thus‐acquired ellipsometric angles *Ψ* and Δ express a relationship between the complex reflectance ratio (*ρ*) and the thin film parameters of thickness (*h_i_*) and complex refractive index ($\tilde{n_{i}}$).^[^ ^43^ ^]^ Since the analytical derivation of $\tilde{n_{i}}$ and *h_i_* is not possible (besides some special cases), iterative optical modeling (initial guess for $\tilde{n_{i}}$ and *h_i_* and forward calculations) with goodness of fit optimization is routinely used for data acquisition from ellipsometry spectra.

As an extension of in situ SE, ellipsometric porosimetry (EP) has been developed as a thin film‐specific characterization technique based on a combination of ellipsometry and vapor sorption.^[^ ^44^ ^]^ EP is utilized to obtain and evaluate volume adsorbed isotherms through optical means, rather than via microgravimetry as in the case of traditional vapor adsorption. Volume adsorbed (*V_ads_*/∑*V* ^0^) is defined as the ratio of condensed phase adsorptive volume and the summed volume of sorbent, sorptive, and void. The latter is constant during the entire pore filling process, because an increase in condensed sorptive causes the same decrease in void.

This study aims to enhance the understanding of vapor adsorption and absorption in PIMs through experimental guidelines, supported by optical analysis, micropore sorption, and polymer physics models. We explore how EP can characterize key properties such as pore size distribution (PSD) and refractive index changes during sorption. By establishing a methodological framework, this work provides a generalizable approach for distinguishing adsorption and absorption in microporous polymers and other porous materials. Understanding these physical processes have broad implications on the design of porous polymers and thin film membranes for broad applications in gas and chemical separation, sensing, and devices for energy conversion and storage.

## 2 Results and Discussion

**Figure 1:**
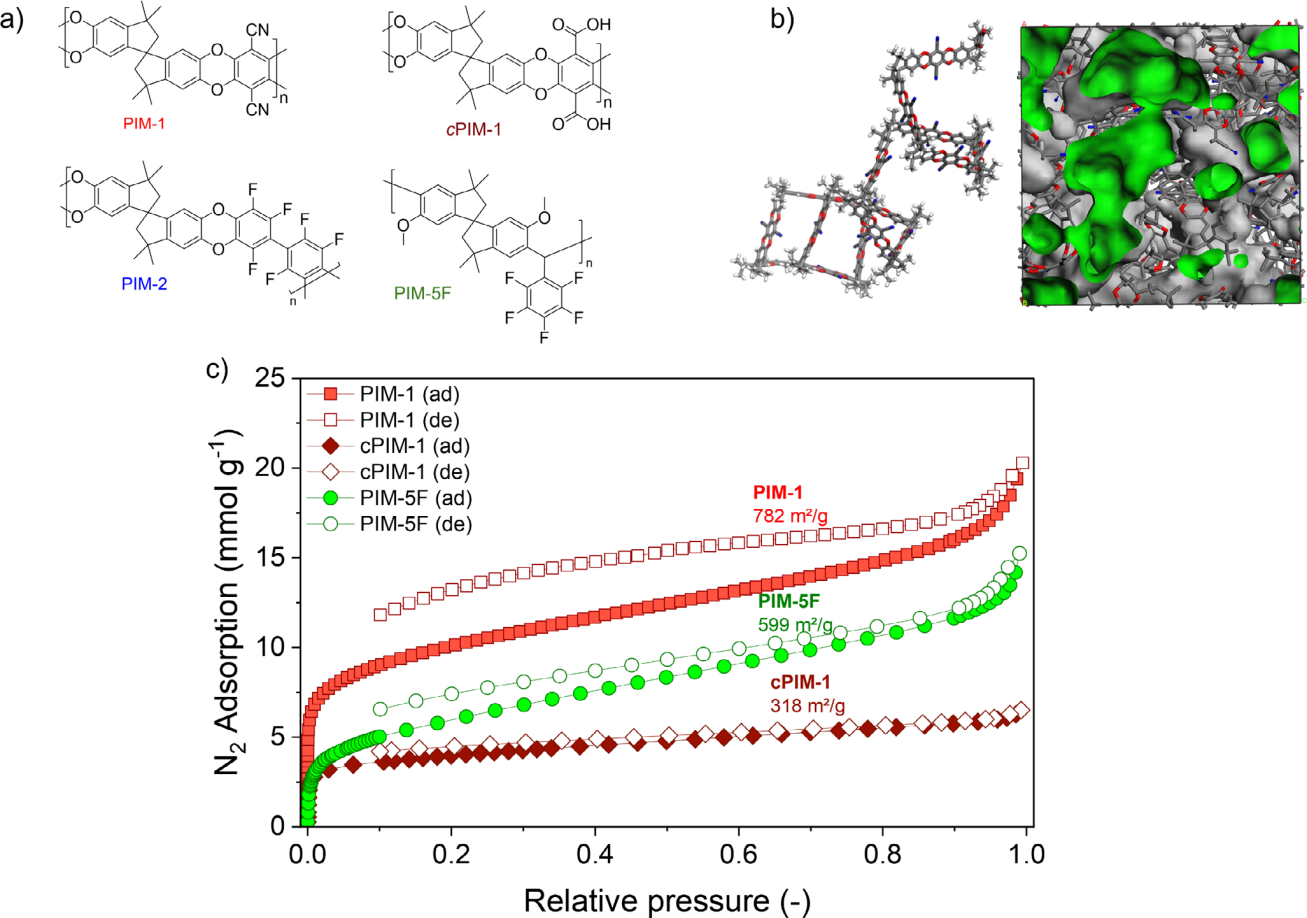
PIM materials with microporosity and tailored polarity. a) Chemical structures of polymers with varied hydrophobicity, including PIM‐1, PIM‐2, *c*PIM‐1, and PIM‐5F. b) Molecular model of a PIM‐1 chain segment with contorted backbone and surface view of one amorphous cell of PIM‐1 (31.8 × 31.8 × 31.8 Å). c) N_2_ adsorption in polymer solids at 77 K.

**Figure 2:**
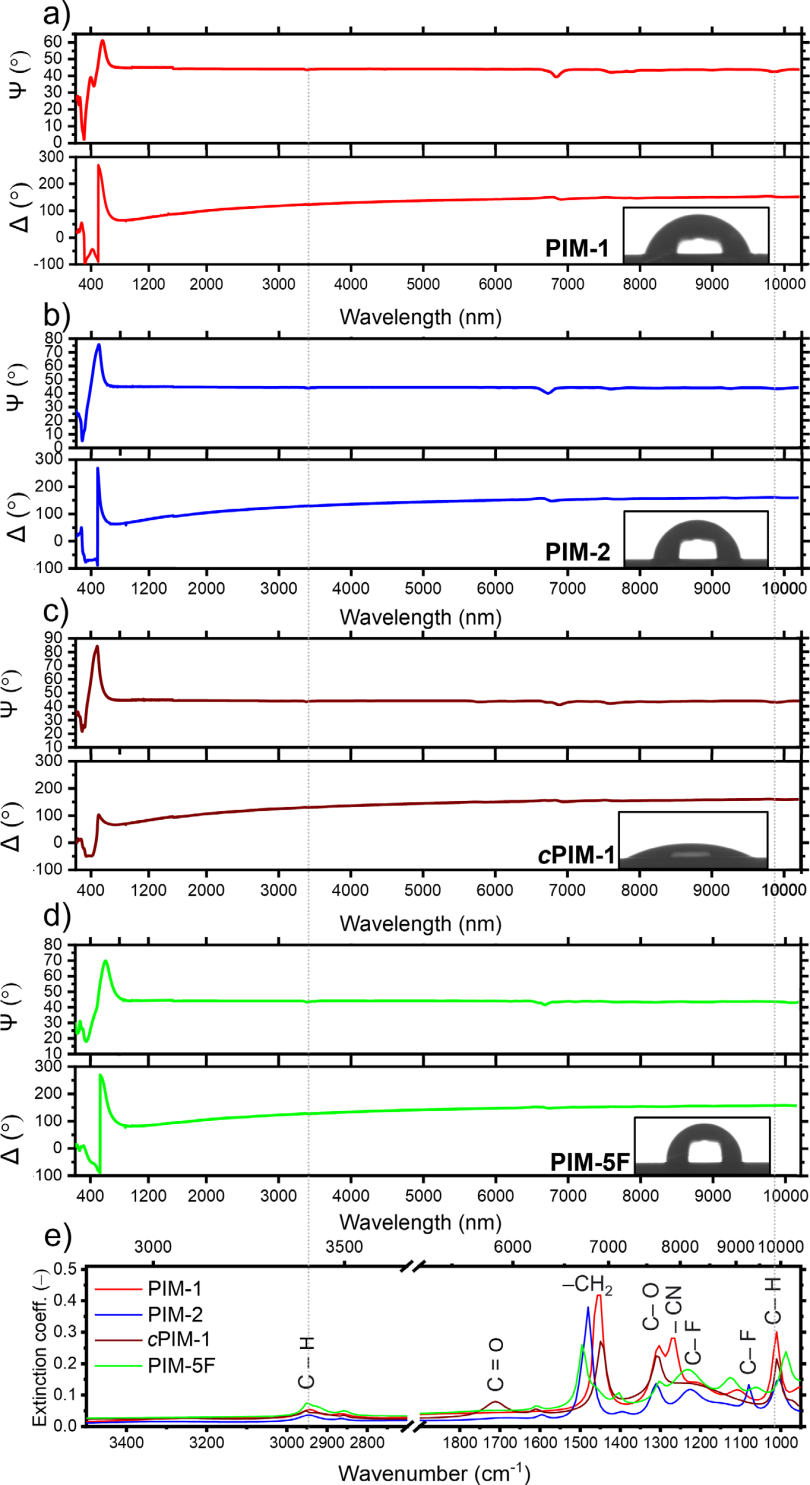
a–d) Full range (UV–vis–IR) ellipsometry spectra (at 75° incidence) of PIM films deposited on Au, with insets of corresponding water droplet shape. e) Fitted mid‐IR extinction peaks and corresponding molecular vibrations. Vertical dotted lines at 2950 cm^−1^ (*λ* = 3390 nm) and 1010 cm^−1^ (*λ* = 9901 nm) corresponding to peaks associated with C–H vibrations serve as guides for the eye.

In this work, PIM thin films of selected material chemistries were investigated using SE and EP to better understand the vapor sorption behaviors associated with pore confinement and swelling. For PIMs, microporosity is considered to comprise the fractional free volume between their contorted chains. Consequently, PIMs allow precise control both over surface hydrophilicity, polarity, and micropore volume via their synthesis route, which has been extensively studied for various applications. For our investigation, we prepared PIM‐1 ‐ the most commonly studied material of its class‐, and carboxylated PIM‐1 (*c*PIM‐1), which exhibit increased hydrophilicity due to the substitution of nitrile with carboxyl functional groups, respectively. In contrast, PIM‐2 and PIM‐5F, containing carbon‐fluorine bonds, were prepared to demonstrate enhanced hydrophobicity. The chemical structures are presented in **Figure** **1**a. Owing to the contorted backbone structures (Figure 1b), PIM polymer chains cannot pack efficiently and form sub‐nanometer‐scale pores, as presented in the molecular model.^[^ ^12^ ^]^ The nitrogen gas sorption at 77 K confirmed the high gas uptake and microporosity of these polymers (Figure 1c). Corresponding droplet shapes are shown in **Figure** **2**, with contact angle values detailed in the supporting information with complementary gravimetric water vapor sorption analysis (Figure S1, Supporting Information).

UV–vis ellipsometry has been thoroughly studied for dense (non‐porous) polymer coatings and was demonstrated to accurately distinguish *n* and *h* variations, particularly within the polymer thickness of 50–2000 nm range when using highly reflective substrates (Si or Au).^[^ ^45^, ^46^, ^47^ ^]^ However, in films thicker than a few microns, interference oscillations tend to dominate the spectra, making it difficult to independently resolve *n* and *h* due to the limited spectral resolution of the detector.^[^ ^46^ ^]^ Conversely, ultrathin films (<40 nm) exhibit low spectral contrast,^[^ ^48^ ^]^ again reducing measurement reliability, which can be mitigated to some degree by tuning the thickness of an underlying SiO_2_ layer on the substrate (i.e., interference enhancement).^[^ ^48^, ^49^ ^]^

Following this recommendation, we performed solution‐processing and spin‐coating steps on both Si and Au‐coated Si substrates to achieve thin films with *h* in the 70–110 nm range. We note that this thickness range is significantly larger than the characteristic pore domain size (<1 nm), which is beneficial for minimizing possible birefringence effects as the model assumes optically random packings of the polymer. Generally, PIMs in this thickness range tend to exhibit more bulk‐like behavior compared to ultrathin films (<40 nm), the optical and swelling properties of which are highly thickness‐dependent.^[^ ^50^ ^]^

Additionally, the wider thin film range of 10–2000 nm is particularly relevant for PIM applications as high‐permeance gas separation,^[^ ^51^, ^52^, ^53^ ^]^ or as a protective layer deposited on a thicker dense/macroporous support,^[^ ^54^ ^]^ where polymer interactions with liquid environments are critical for performance.^[^ ^55^ ^]^ Full‐range (UV–vis–IR) ellipsometry spectra (with all separate measurements and fitted spectra shown in Figures S2 and S3, Supporting Information) are showcased in Figure 2, measured on the investigated PIM thin films deposited on Au. In the UV–vis regions, peaks are associated with refractive index dispersion convoluted with thin film interference oscillations. Since all films were thinner than 110 nm, only a single oscillation peak appears in the vis region of the respective spectra, without extending beyond *λ >* 900 nm. In the analyzed mid‐IR region (3000 nm < *λ <* 10 000 nm), the fitted extinction coefficient peaks can be assigned to absorption bands based on their wavenumber, characteristic of functional groups (Figure 2e). These correspond to stretching bonds of PIM chemistry (while being unrelated to *h*) and give information on the expected polarity of the polymer (i.e., C─N and C═O stretching indicating increased polarity and hydrophilicity, while C–F stretching suggests the contrary behavior).

Since the spectral features related to *h* are prominent in the UV–vis region, in situ monitoring of structural changes during adsorption (indicated by an increase in *n*, or optical density) and absorption (indicated by a simultaneous increase in *h* and decrease in *n*) processes is relatively straightforward using vacuum EP equipment with controlled atmosphere. Ellipsometric read out of relative pressure‐dependent change in sorbent thin films offers several advantages over other optical techniques like UV–vis reflectometry and interferometry. Ellipsometry is particularly sensitive and selective to changes in *n* and *h*, in contrast to other optical techniques that are primarily sensitive to the optical path length (n*h) and can less reliably differentiate between these two key parameters. In particular, the phase shift information (Δ) acquired by rotating compensator ellipsometry improves selectivity and sensitivity over conventional optical measurements using unpolarized light.^[^ ^43^ ^]^

**Figure 3:**
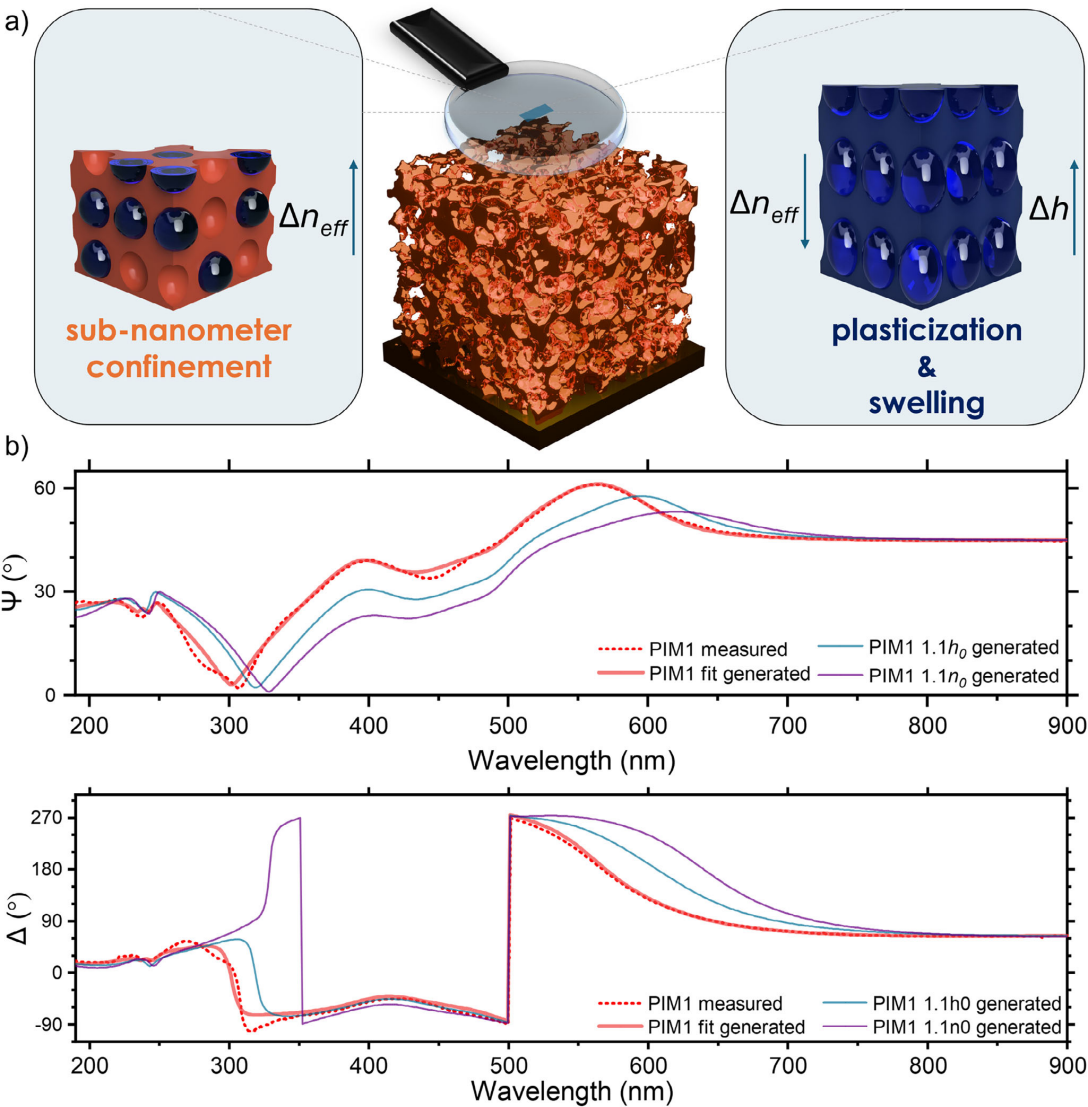
a) Schematic of nanopore confinement and swelling as rival modes of vapor uptake in PIMs, and their opposing effect on the optical density of the film. b) Measured and fitted UV–vis ellipsometric spectra (at 75° incidence) of thin PIM‐1 film on Au, complemented by simulated cases for 10% increase in thickness and refractive index, respectively.

This is demonstrated in **Figure** **3**, which shows two simulated ellipsometric spectra of PIM‐1—one with a 10% increase in *h* and the other with a 10% increase in *n*. The resulting ellipsometric *Ψ*‐Δ spectra are clearly distinct, despite the films having identical optical path lengths across the full vis wavelength range, providing support for the case of ellipsometric analysis as the tool for the envisioned full disentanglement of adsorption and absorption effects. We note that the good description of the optical behavior by our isotropic model suggests low to no birefringence effects, which is in line with a previous study of PIM materials with similar thickness.^[^ ^56^ ^]^

**Figure 4:**
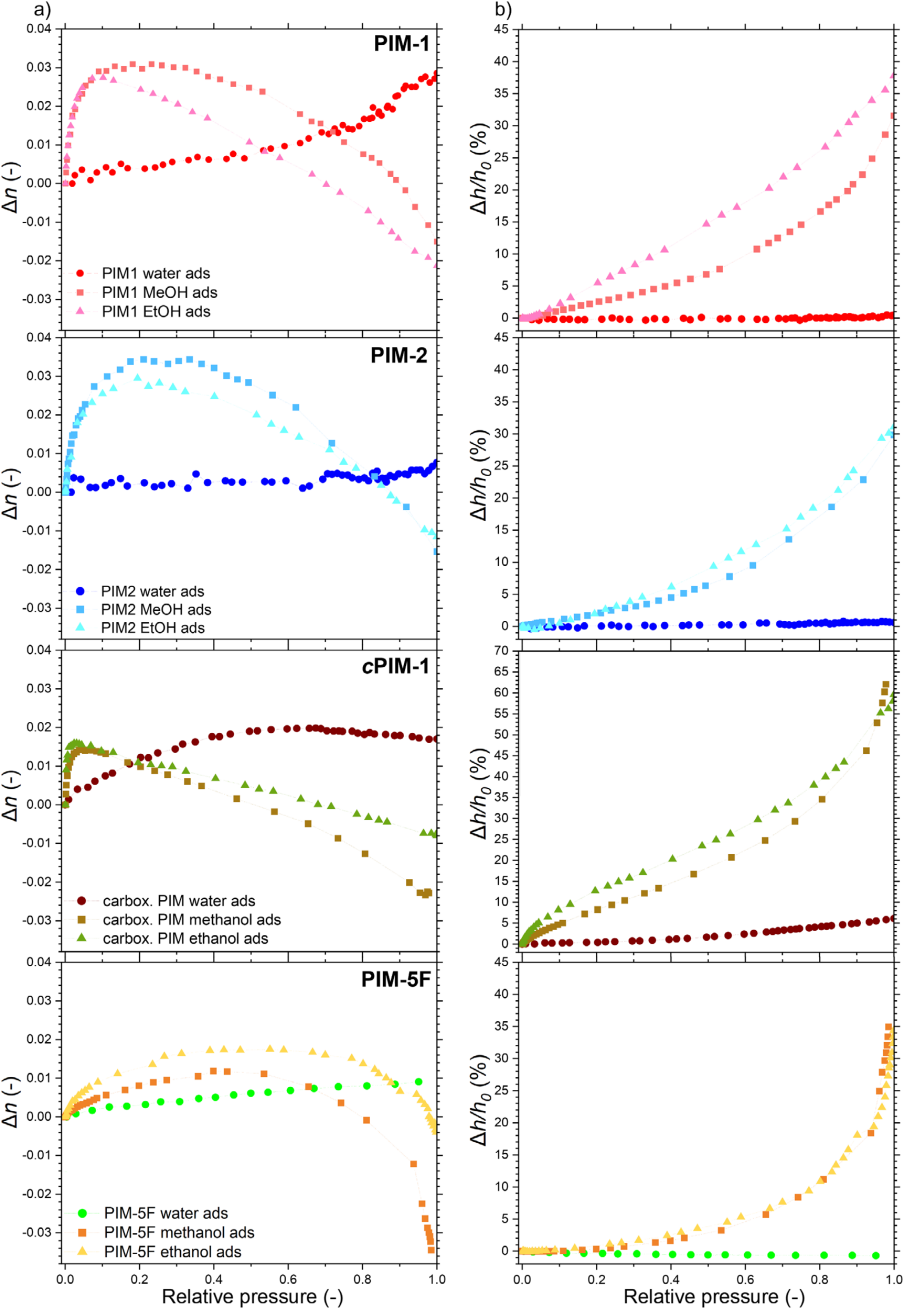
PIM *n* isotherms a) and *h* isotherms b) for water, methanol, and ethanol (increasing relative pressure only, *n* taken at *λ* = 632 nm).

All sorption‐based characterization techniques produce isotherms as their primary measurement output. These isotherms quantify the uptake of a fluid sorbate in the studied sorbent and may be obtained from changes in pressure (volumetric methods) or variations in sorbent mass (gravimetric methods). In the case of EP, the raw output is the relative pressure‐dependent variations of *Ψ*‐Δ spectra (optical method). Using an optical model with high goodness of fit (such as the one shown in Figure 3b), this data can be converted into *n* and *h* isotherms, as illustrated in **Figure** **4**. (with desorption isotherms provided in the Figures S4–S7, Supporting Information).

More specifically, Figure 4a graphs present changes in *n* at *λ* *=* 632 nm. The refractive index increases during adsorption due to the material's densification during micropore filling. As the relative pressure (*P/P^0^*) rises, the material undergoes swelling (suggesting that the polymer matrix expands). This has a visible impact on *n*, as the material's optical density declines. This finding reinforces the micropore adsorption mechanism, which infers that after pore‐filling, additional adsorbate accumulates on the polymer matrix, thereby leading to swelling. The EP data reveals significant trends exhibited by PIMs when exposed to methanol, ethanol, and water vapors. All PIM samples exhibit an increase in *h* upon *P/P^0^* increase (except for water, which cannot penetrate hydrophobic matrices), with *c*PIM‐1 showing the most pronounced change (≈60% increase for methanol and ethanol). Furthermore, *c*PIM‐1 is the only investigated polymer that undergoes observable swelling in high *P/P^0^* water due to the hydrophilic nature of its matrix. We note that *h* decreases in the initial parts of some of the isotherms due to the effect of adsorption‐induced deformation (AID) of the porous structure (see detailed discussion in the Supporting Information),^[^ ^57^, ^58^, ^59^, ^60^, ^61^, ^62^ ^]^ although this phenomenon is much less pronounced than the subsequent (macroscopic) matrix swelling. The sections on the isotherms where adsorption and swelling occur respectively, are identifiable: it simply requires determining whether *n* is increasing or decreasing at a specific *P/P^0^*. The decrease in *n* is also accompanied by an increase in *h*, which confirms the underlying mechanism.

We note that the measurement time for EP cycles was deliberately kept short (<15 min), to avoid time‐dependent plasticization effects, thereby isolating primary adsorption and swelling phenomena, which occur on much faster time scales.^[^ ^50^ ^]^ The magnitude of vapor‐induced plasticization (or aging) effect is more significant at higher *P*/*P* ^0^, where the partial transition of the glassy polymer to rubbery state is induced. However, it was proven that such secondary effects take hours‐to‐days for effectively reaching equilibrium, even in thin films.^[^ ^50^, ^63^, ^64^ ^]^ To show that the effect of aging is minimal at rapid measurements, we conducted timed measurements at a certain relative pressure, which resulted in negligible time‐dependent thickness change in a 2 h timeframe (see Figure S9, Supporting Information).

In the next section, the observed changes in *n* and *h* are used to calculate volume ad/absorbed contributions and obtain disentangled isotherms. For subsequent operations with the Bruggeman effective medium approximation (EMA), the film refractive index dispersion in the visible wavelength range, and the adsorptives’ known refractive indices were used. Generally, with 3‐component EMA mixing, the effective complex refractive index (${\tilde{n}}_{eff}$) is expressed the following way by the volume fractions *V* of the sorbent, sorptive and void pockets respectively.

(1) $$\begin{array}{ccc} & & V_{\mathrm{sorb}\mathrm{ent}}\frac{{\left({\tilde{n}}_{\mathrm{sorb}\mathrm{ent}}\right)}^{2}-{\left({\tilde{n}}_{\mathrm{eff}}\right)}^{2}}{{\left({\tilde{n}}_{\mathrm{sorb}\mathrm{ent}}\right)}^{2}+2{\left({\tilde{n}}_{\mathrm{eff}}\right)}^{2}}+V_{\mathrm{sorp}\mathrm{tive}}\frac{{\left({\tilde{n}}_{\mathrm{sorp}\mathrm{tive}}\right)}^{2}-{\left({\tilde{n}}_{\mathrm{eff}}\right)}^{2}}{{\left({\tilde{n}}_{\mathrm{sorp}\mathrm{tive}}\right)}^{2}+2{\left({\tilde{n}}_{\mathrm{eff}}\right)}^{2}}\\ & & +V_{\mathrm{void}}\frac{1-{\left({\tilde{n}}_{\mathrm{eff}}\right)}^{2}}{1+2{\left({\tilde{n}}_{\mathrm{eff}}\right)}^{2}}=0 \end{array}$$

(2) $$\begin{array}{c} V_{\mathrm{sorb}\mathrm{ent}}+V_{\mathrm{sorp}\mathrm{tive}}+V_{\mathrm{void}}=1 \end{array}$$

**Figure 5:**
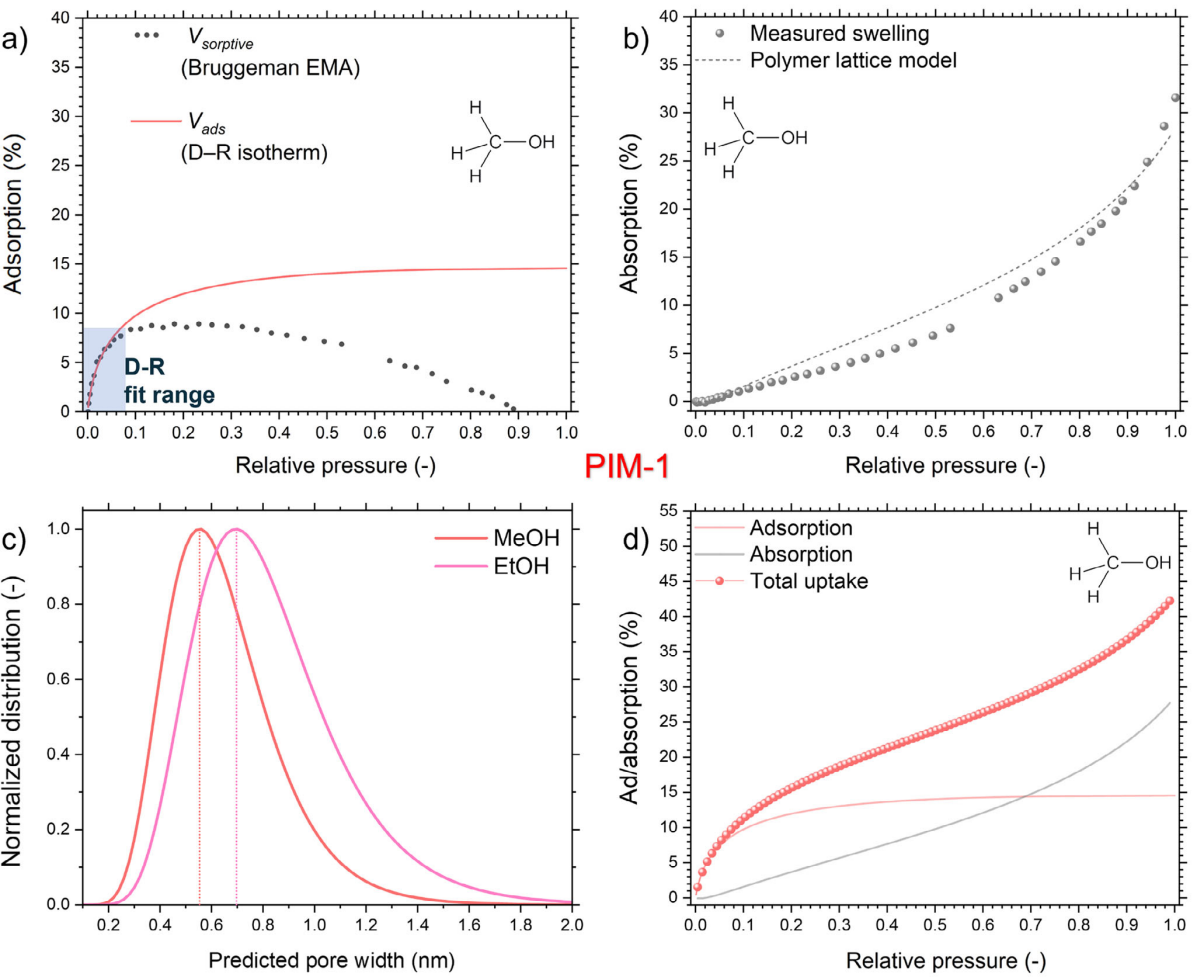
PIM‐1 methanol adsorption a) and absorption b) disentangled. c) Pore width distribution from D–R model. d) Total vapor uptake calculation based on superposition of the isotherms.

The calculation was performed separately for each relative pressure step. As a demonstration of the method, the result of 3‐component Bruggeman EMA fit is showcased in **Figure** **5**a for PIM‐1 methanol isotherm. Due to matrix swelling, this model is not usable at *P/P^0^* values higher than 0.2 in the shown case, as the *V_sorptive_* isotherm is seemingly non‐monotonic. This *V_sorptive_* decrease (calculated from *n_eff_* decrease) is an artifact of fitting procedure, which relates to the declining refractive index of the polymer matrix itself during swelling. This effect cannot be taken into account in the EMA model without introducing high parameter correlation, which would reduce the reliability of the acquired *V_sorptive_* values in the non‐swelling regions of the *n* isotherms as well (see detailed explanation in the Supporting Information).

Thus, the Bruggeman EMA could not be applied in the swelling region. To alleviate this artifact, we fitted the initial part of the *V_sorptive_* isotherm with the Dubinin–Radushkevich (D–R) isotherm (see Figure 5a). The D‐R model was originally developed for describing vapor sorption in microporous carbons^[^ ^65^ ^]^ and has since seen usage for other functionalized sorbents such as organosilicates^[^ ^66^ ^]^ and other low‐k dielectrics.^[^ ^67^ ^]^ Not only does the D–R model exhibit good fit quality, but it can also be extrapolated to the full *P/P^0^* range to serve as a volume adsorbed (*V_ads_*) isotherm which disregards any absorption effects. Thus, a volume adsorbed isotherm can be obtained based on the aforementioned optical model combined with the D–R‐fit. We note that for reliable analysis with the D–R model, low‐to‐no swelling effects need to be present in the fitted region of the isotherm.

For following suit in acquiring the corresponding volume absorbed isotherms using our data, we first consider the thickness change to be solely indicative of swelling. Thus, we readily convert swelling to volume absorption.

(3) $$\begin{array}{c} \frac{V_{\mathrm{abs}}}{\sum V^{0}}∼\frac{\Delta h}{h_{0}} \end{array}$$

A more detailed description of this relation (based on polymer lattice theory) is shown in the Supporting Information. The experimental swelling isotherms were fitted with the model to gain a better understanding of the materials chemistry effect on absorption behavior (see Figure 5b). Within our model, physical adsorption was assumed to occur uniformly in the micropores, while gas molecules can also be absorbed into the polymer matrix. These processes are accompanied by deformation: adsorption causes adsorption‐induced deformation (schematic in Figure S12, Supporting Information), and absorption leads to polymer swelling. AID is modeled as a change in pore volume in the low‐pressure adsorption isotherms, independent of swelling (see detailed discussion and fits in Figures S13–S15, Supporting Information).

The pore width distribution obtained from methanol adsorption via the D‐R fit (see Figure 5c)–based on the correlation of the fitted heat of adsorption and micropore size (Equations S18–S21, Supporting Information)–in case of methanol is remarkably similar to expectations from previous molecular simulations.^[^ ^31^ ^]^ As shown, ethanol sorption slightly overestimates pore sizes, which is due to the larger molecule size of this sorptive. We note that methanol was previously selected as the preferred EP adsorptive for micropore sorption.^[^ ^68^, ^69^ ^]^ From the disentangled adsorption and absorption contributions, we can further predict total vapor uptake isotherms via Equation (4), expressed in volume%.

(4)

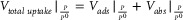




**Figure 6:**
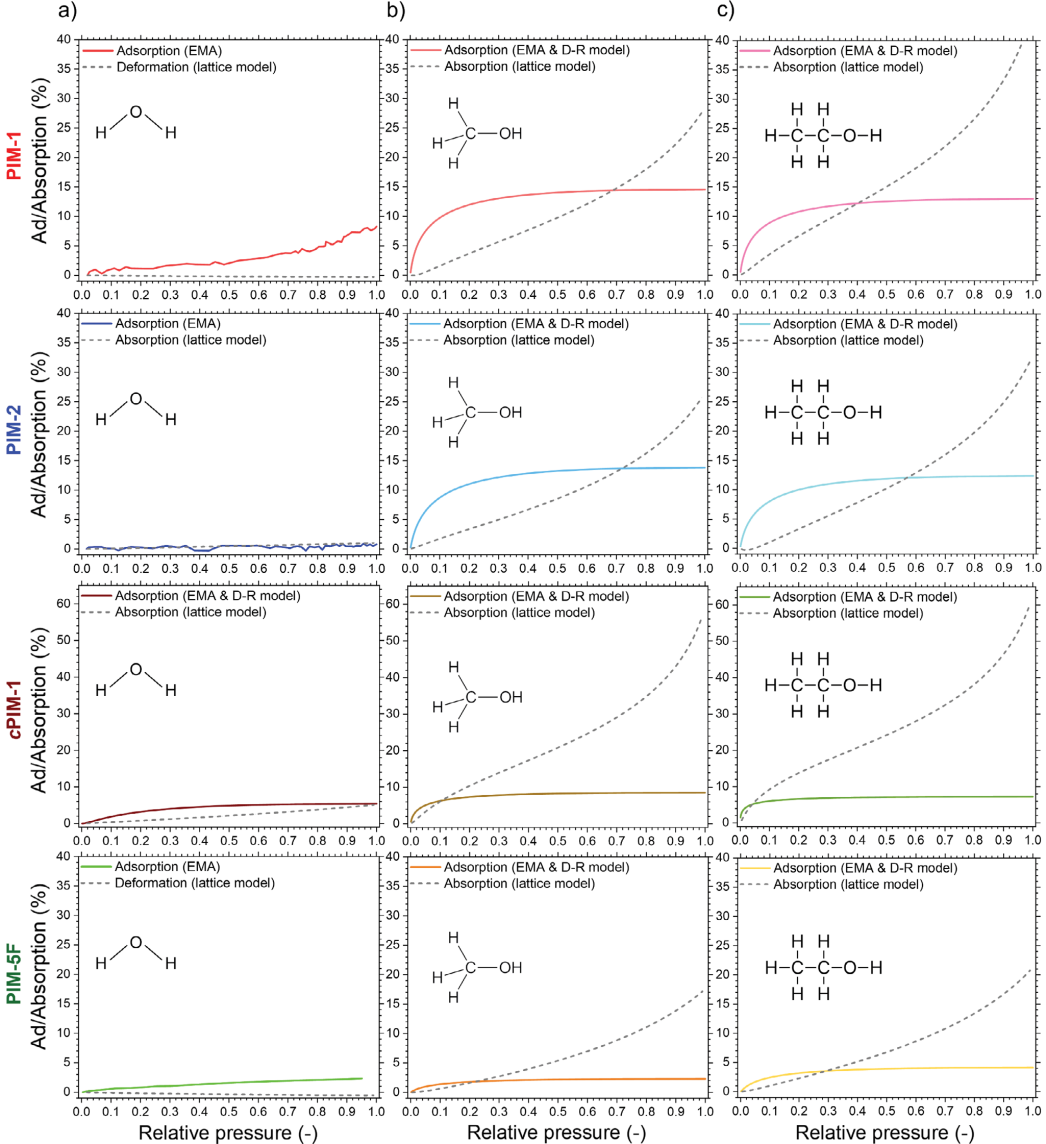
Disentangled adsorption/absorption (solid/dashed lines) contributions for all polymer‐sorptive pairings with fitted models (the three columns correspond to water, methanol and ethanol sorption). Note that PIM‐1 and PIM‐5F water sorption contain only deformation and adsorption components.

To expand our analysis to all PIM‐adsorptive pairs studied, **Figure** **6** showcases the separately resolved adsorption and absorption contributions, with the three columns corresponding to water, methanol and ethanol sorption. As a result of our analysis, the fitted D–R isotherms (adsorption) and polymer lattice model‐based‐isotherms (absorption) are plotted, except in cases where the D–R isotherm is not appropriate (water adsorption in the more hydrophobic PIMs).

Key aspects of the PIMs’ porous structures are revealed in Figure 6. For the alcohol uptake of moderately polar PIM‐1 and PIM‐2, the regions where adsorption and absorption respectively, dominate, are identifiable. We also see that water uptake is exclusive to adsorption in PIM‐1, and negligible for both adsorption and absorption in PIM‐2. These results agree well with those measured by gravimetric sorption technique and are in good correlation with the expected effects on increasingly apolar materials chemistry of these PIMs. Meanwhile, the highly polar *c*PIM‐1 undergoes over 60% swelling in methanol vapor due to hydrogen‐bonding between alcohols and its polymer matrix. Nonetheless, its uptake capacity via adsorption is significantly reduced compared to PIM‐1 (≈15% vs 8%). This observation is in‐line with specific surface area analysis (reported as 782 vs 318 m^2^g^−1^) measured by N_2_ adsorption (Figure 1c)^[^ ^70^ ^]^ of these particular PIM structures and highlights a decrease in relative pore volume as a result of bulkier carboxyl groups. PIM‐5F on the other hand, shows a decreased capacity for both adsorption and absorption of alcohols, which is explained by its severely apolar composition. We once again highlight the importance of adsorptive molecular size for estimating uptake capacity by adsorption (and thus relative pore volume). As shown in Figure 6, all investigated PIMs adsorb more methanol compared to ethanol, except for the hydrophobic PIM‐5F, which exhibits the opposite behavior. We attribute this to the surface chemistry of the C─F groups favoring the more apolar ethanol molecules. In the other three cases, however, some ethanol molecules might be excluded from entering the smallest ultramicropores, whereas methanol can still access them. This interpretation is further supported by the deviations in pore size distribution derived for PIM‐1 by methanol and ethanol respectively (Figure 5c).

**Figure 7:**
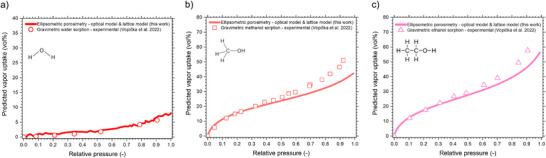
Validation of EP‐modeled isotherms. a–c) Predicted total vapor (water, methanol, ethanol) uptake of PIM‐1 based on EP results analyzed with EMA and lattice‐modeling approach (full *V_ads_* + *V_abs_* contributions, represented by solid lines), compared to gravimetrically measured vapor uptake of PIM‐1 powders (source of gravimetric data: Vopička et al. 2022).^[^ ^34^ ^]^

The three total vapor uptake isotherms obtained for PIM‐1 are presented in **Figure** **7**. PIM‐1–being the first reported polymer of its class–is frequently studied in the literature using gravimetric methods,^[^ ^34^ ^]^ making it an excellent benchmark for comparing our modeled isotherms. As a comparison to the ellipsometric acquisition of *V_ads_* + *V_abs_* isotherms (based on the analysis exhibited in Figure 5), gravimetric isotherms measured by previous authors^[^ ^34^ ^]^ of PIM‐1 are plotted. As shown, the shapes of the uptake isotherms closely resemble those of experimentally measured gravimetric uptake isotherms. Gravimetric sorption is known for producing highly reliable vapor uptake data on powdered materials, as it directly measures the combined ad‐/ and absorbed mass, providing a strong reference for our indirect optical approach.

On the other hand, our alternative optical approach based on EP supplies further insights into sorption, as expressed by the aforementioned *n* and *h* isotherms, which also allows us to predict pore volume, swelling and vapor uptake isotherms through fitting. The close similarity between EP‐predicted vapor uptake and experimental gravimetric uptake strongly validates the models used in this study, starting from the fitting of *Ψ*‐Δ spectra and subsequent modeling with the D–R isotherm and the polymer lattice model. We theorize that the consistently smaller predicted uptake by EP (compared to gravimetric sorption) at *P*/*P* ^0^ > 0.7 for methanol and ethanol could be caused by the rapid EP measurement times, which were used to minimize plasticization effects and aging. We note that further minor differences can also be caused by the inherent differences of the sorbent structure (thin films as opposed to powdered solid). Remarkably, despite UV–vis ellipsometry analyzing ≈1 × 1 mm spots of <100 nm polymer films—corresponding to less than 100 ng of actively analyzed PIM material—the resulting isotherms closely align with those obtained from gravimetric measurements on macroscopic PIM samples.

## 3 Conclusion

In summary, this study demonstrates how ellipsometric readout can effectively distinguish between adsorption and absorption processes in microporous polymers. By leveraging refractive index and thickness isotherms, we developed a systematic approach to analyze vapor sorption behavior and disentangle adsorption and absorption mechanisms.

Our modeling framework, combining the three‐component Bruggeman effective medium approximation and Dubinin–Radushkevich‐predicted isotherms, provided not only a strong fit to experimental data but also valuable structural insights, including free pore volume, pore size distribution, and sorptive affinities. We further established the critical role of PIM chemistry in governing both microporosity and interactions with different adsorptives, as evidenced by variations in swelling behavior across different PIMs.

The analytical approach outlined in this study extends beyond PIMs and offers a generalizable methodology for characterizing vapor sorption in a broad range of nanoporous soft materials. These include biological materials, organic‐inorganic hybrids, polymer membranes, and framework materials such as metal‐organic and covalent‐organic frameworks. Future works are anticipated in studying the dynamics of swelling/plasticization with the described data analysis tools, complemented by in situ atomic force microscopy, X‐ray reflection, and Mueller matrix ellipsometry for the full description of anisotropic behavior of such systems.

Such improved understanding of sorption behavior provides a basis for designing high performance materials tailored to gas separation, filtration, sensing, and energy conversion and storage.

## 4 Experimental Section

Detailed sample preparation and synthesis procedures were elaborated in the Supporting Information. We note that for independent analysis of all sorbent‐sorptive pairs, separate spin‐coated thin film samples were used for measurements with all separate adsorptives.

### Ellipsometry and Ellipsometric Porosimetry Measurements

Ellipsometric porosimetry experiments were carried out using Semilab SE‐2000 rotating compensator spectroscopic ellipsometer with a vacuum chamber extension. Measurements were performed at an incidence angle of 70°, with sample temperature at 36 °C with rapid sorption cycles (< 15 min.) at the wavelength range of 277–989 nm (UV–vis). The relative vapor pressure of the selected volatile adsorptives was controlled with a proportional valve connected to the vacuum chamber, while the pore filling was monitored in situ via spectroscopic ellipsometry measurements. Supplementary IR measurements were performed (ex situ) with an FTIR‐based Semilab ellipsometer at the wavelength range of 1.6–12 µm. All measurement data were analyzed with the ‘Semilab SEA’ software.

Thickness measurements (by ellipsometry) on spin‐coated samples were carried at the center point of the samples and were repeated for three different samples deposited from the same polymer solution (see Figure S8, Supporting Information). Reproducibility of the ellipsometric porosimetry measurements is shown in the Supporting Information.

### Adsorption Model

For modeling microporous adsorption, the Dubinin–Radushkevich (D–R)‐model was used.

The D–R model is grounded in the Polanyi theory of potential adsorption.^[^ ^71^ ^]^ The main driving forces for adsorption within these low relative pressure systems relate to the forces that exist between the pore walls and the sorptive molecules.^[^ ^72^, ^73^ ^]^ Thus, the adsorptive level increase can usually be described by an exponential function. This theory has become known as the volumetric filling of pores, and can be modeled with the D–R isotherm shown below^[^ ^74^ ^]^:

(5) $$\begin{array}{c} V_{\mathrm{ads}}=V_{t}\exp\left\{-\frac{1}{{\left(\beta E_{0}\right)}^{2}}{\left[RT\;\ln\left(\frac{P}{P^{0}}\right)\right]}^{2}\right\} \end{array}$$

where *V_t_* is the total accessible micropore volume. *E*_0_ is the characteristic energy of adsorption for a specific micropore size, while β is purely a sorptive‐related, microporous affinity parameter. The backbone of the D–R model lies in linear regression fitted to the natural logarithm of the volume adsorbed isotherm. Plotting ln(*V_ads_*) against ${\left[\ln(\frac{P}{P^{0}})\right]}^{2}$ yields a linear relationship, which were fitted to the experimental values after conversion. *V_t_* was found from the intercept, while β*E*_0_ (isosteric heat of sorption) has a proportional relationship to the slope. The derivation steps of pore sizes from the isotherms are detailed in the Supporting Information (Equations S18–S21, Supporting Information).

### Gravimetric to Volumetric Conversion

Literature gravimetric data was converted to vol% using the liquid density of the adsorptive (water, methanol, and ethanol). The density of PIM‐1 was assumed to be 1 g cm^−3^.

## Conflict of Interest

The authors declare no conflict of interest.

## Supporting information



Supporting Information

## Data Availability

The data that support the findings of this study are available from the corresponding author upon reasonable request.
