# Supplementary material for: Disentangling Adsorption and Absorption in Microporous Polymers
Source: Small Methods. 2025 Aug 7;9(9):e00845. doi: 10.1002/smtd.202500845 (PMC12464648; doi:10.1002/smtd.202500845)
Supplement: Supplementary file 1 — Supporting Information [file SMTD-9-e00845-s001.pdf]

small methods

Supporting Information

for *Small Methods*, DOI 10.1002/smtd.202500845

Disentangling Adsorption and Absorption in Microporous Polymers

*Máté Füredi, Andrei Kolesnikov, Anqi Wang, Klara Burdova, Natan Abelian, Sunshine Iguodala, Bálint Fodor, Gennady Y. Gor, Qilei Song and Stefan Guldin**

Supporting Information for

Disentangling Adsorption and Absorption in Microporous Polymers

Máté Füredi,[1,2] Andrei Kolesnikov,[3] Anqi Wang,[4] Klara Burdova,[1] Natan Abelian,[1] Sunshine Iguodala,[4] Bálint Fodor,[2] Gennady Y. Gor,[3] Qilei Song,[4] Stefan Guldin[1,5,6*]

[1]*Department of Chemical Engineering, University College London, Torrington Place, London, WC1E 7JE, UK*

[2] *Semilab Co. Ltd., Prielle Kornélia u. 2. H-1117 Budapest, Hungary*

[3]*Otto H. York Department of Chemical and Materials Engineering, New Jersey Institute of Technology, University Heights, Newark, NJ, 07102, USA*

[4]*Department of Chemical Engineering, Imperial College London, London, SW7 2AZ UK*

[5]*Department of Life Science Engineering, Technical University of Munich, 85354 Freising, Germany*

[6]*TUMCREATE, 1 CREATE Way, #10-12 CREATE Tower, 138602, Singapore*

**E-mail address:* s.guldin@ucl.ac.uk, guldin@tum.de

Polymer synthesis procedures

PIM-1

5,5′,6,6′-Tetrahydroxy-3,3,3′,3′-tetramethyl-1,1′-spirobisindane (TTSBI) (3.40 g, 10.0 mmol) and 2,3,5,6-tetrafluoroterephthalonitrile (TFTPN) (2.00 g, 10.0 mmol) were added to a 250 mL three-neck-round-bottomed flask and pre-flashed with nitrogen, before being dissolved in anhydrous dimethylacetamide (DMAc) (18.0 mL). Once a clear solution formed, the flask was placed under reflux at 150˚C, followed by adding K_2CO_3 fine powder (3 x 1.17 g, 25.4 mmol) over 5 minutes, where the resulting solution increased significantly in viscosity over 30 minutes. Subsequently, three portions of toluene (3 x 2 mL) were added over 10 minutes, and the solution was left to stir for a further 1 h. The polymer solution was poured into methanol (200 mL) and left to stir for several hours. After filtering and drying overnight, the polymer solid was dissolved in chloroform and reprecipitated in methanol at least twice. Finally, the polymer was refluxed in water overnight to remove any residual salts and dried at 110˚C overnight to give PIM-1 as a yellow powder at 94% yield.

PIM-2

TTSBI (2.04 g, 6.0 mmol) and K_2CO_3 (0.83 g, 6.0 mmol) were dissolved in a three-neck-round-bottomed flask equipped with a reflux condenser under a nitrogen atmosphere. 12 mL anhydrous dimethylformamide (DMF) was added using a syringe and needle, and the mixture was allowed to dissolve at 100 °C for 30 minutes. Decafluorobiphenyl (2.00 g, 6.0 mmol) was dissolved in anhydrous DMF (58 mL) in a separate round-bottomed flask kept at room temperature. It was transferred into the first reaction mixture drop-wise using a syringe over 3 h. The mixture was left to react for 48 h at 100 °C. The white suspension was poured into 150 mL of aqueous HCl (5%) under constant stirring, and the precipitate was filtered under a vacuum. The crude product was dried at 120 °C overnight, washed with methanol and dried again overnight at 100 °C, to give PIM-2 as white powder at 76% yield.

cPIM-1

To a 1 L flask containing PIM-1 (10 g) as a fine powder, deionized water (250 mL), glacial acetic acid (83 mL) and concentrated sulfuric acid (250 mL) were added sequentially, and placed under reflux at 150˚C for 48 h. The suspension was then cooled, and vacuum filtered directly. The powder was thoroughly washed with deionized water, while the pH was monitored continuously. It was then transferred to a 1 L round-bottom flask containing 0.1 M aqueous sulfuric acid (500 mL) and heated to reflux overnight. The powder was then filtered, washed with abundant deionized water and dried overnight at 110˚C to give cPIM-1 as a dark orange powder at 95% yield.

PIM-5F

Firstly, bisphenol A (200 g) was melted at 140 °C, followed by the addition of methanesulfonic

acid (10g). The mixture was reacted for 5 h with mechanical stirring, and then slowly poured into ice water. The precipitate was washed three times with water and recrystallized using ethanol as a solvent, to result in white needle-like crystals of dihydroxyspirobisindane (SBI-OH). SBI-OH (4.63 g, 15.00 mmol) was transferred to a 100 mL three-neck round-bottomed flask and pre-flashed with nitrogen, before being dissolved in anhydrous DMF (50 mL) at room temperature. K_2CO_3 (8.32 g, 60.20 mmol) was then added in one portion. Following this, iodomethane (2.40 mL, 39.00 mmol) was added dropwise to the mixture. After stirring overnight, the mixture was poured carefully into water. The precipitate obtained was washed with water several times, followed by filtering and drying overnight at 110°C to give dimethoxyspirobisindane (SBI-OMe) at 92% yield.

In the following procedure in a 50 mL three-neck-round-bottomed flask, 2,3,4,5,6-Pentafluorobenzaldehyde (3.92 g, 20 mmol) and SBI-OMe (6.72 g, 20 mmol) were dissolved in dichloromethane (DCM) (10 mL). Methanesulfonic acid (6.50 mL) was promptly added in one go at room temperature while stirring continued. At the end of the polycondensation, after a significant change in viscosity was observed, the reaction mixture was precipitated into ammonia solution. The polymer solid was collected, dissolved in a small amount of DCM, and precipitated into methanol. This was repeated at least three times. The white polymer was then filtered and dried under vacuum at 60 °C overnight to produce PIM-5F at 90% yield.

Thin film deposition

Thin films of the synthesized polymers dissolved in solvents (PIM1, PIM2, and cPIM-1: 1% in chloroform; PIM-5F: 1% in tetrahydrofuran) were obtained using a Laurell WS 650 MZ spin-coater. Spin-coating were carried out at 5000 rpm for 60 seconds. As substrates, 1x1 cm Si wafer cuts and gold-coated Si wafer cuts (Sigma Aldrich) were used respectively for EP measurements and ex-situ SE measurements (UV-Vis, NIR and IR). The substrates were cleaned in oxygen plasma prior to film deposition.

Drop shape analysis

Water contact angles were recorded using a Kruss DSA100 drop-shape analyzer. Test liquid droplets of ~5 µl were deposited on the studied PIM surfaces and contact angle values were calculated from the shapes of the three-phase contact lines

Contact angle determination and gravimetric water sorption

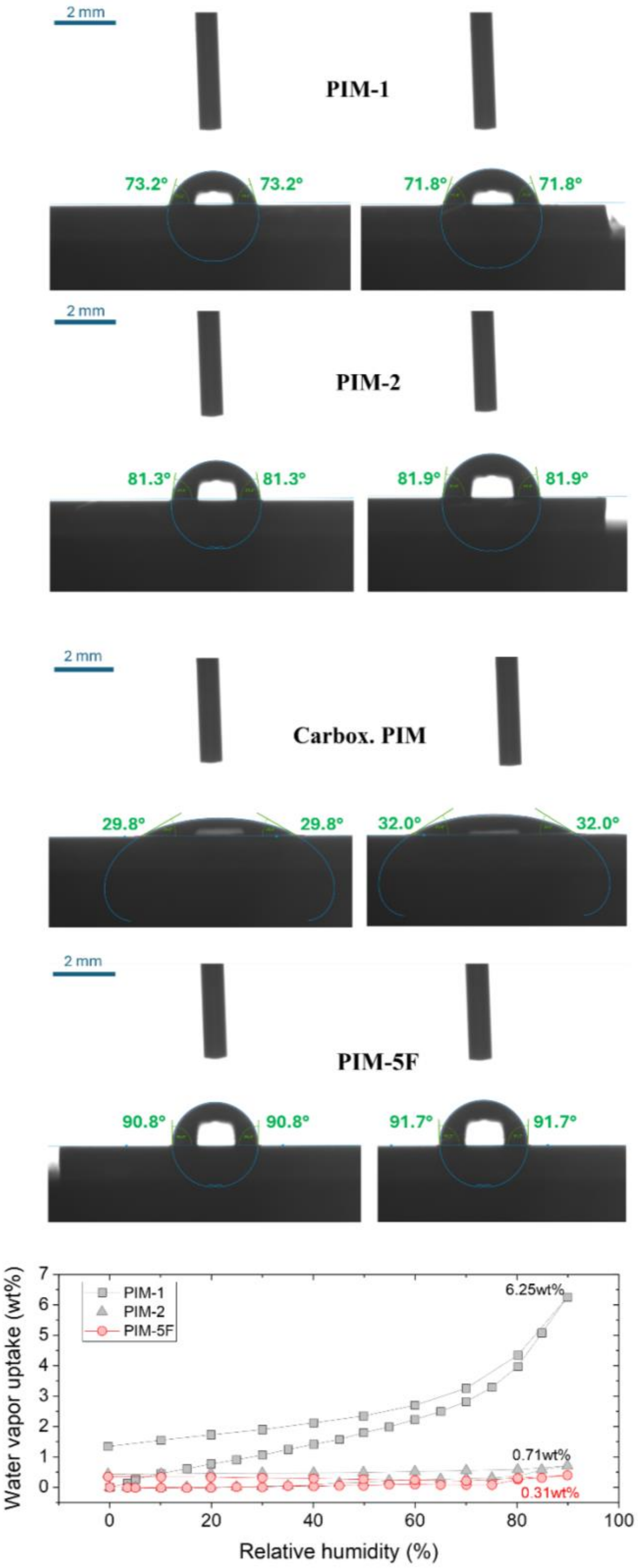

Figure S1. Water droplet shapes and fitted water contact angles on PIM thin films (2 separate droplets measured for each film) -top. Gravimetric water sorption – bottom.

Ellipsometry spectra and fitting

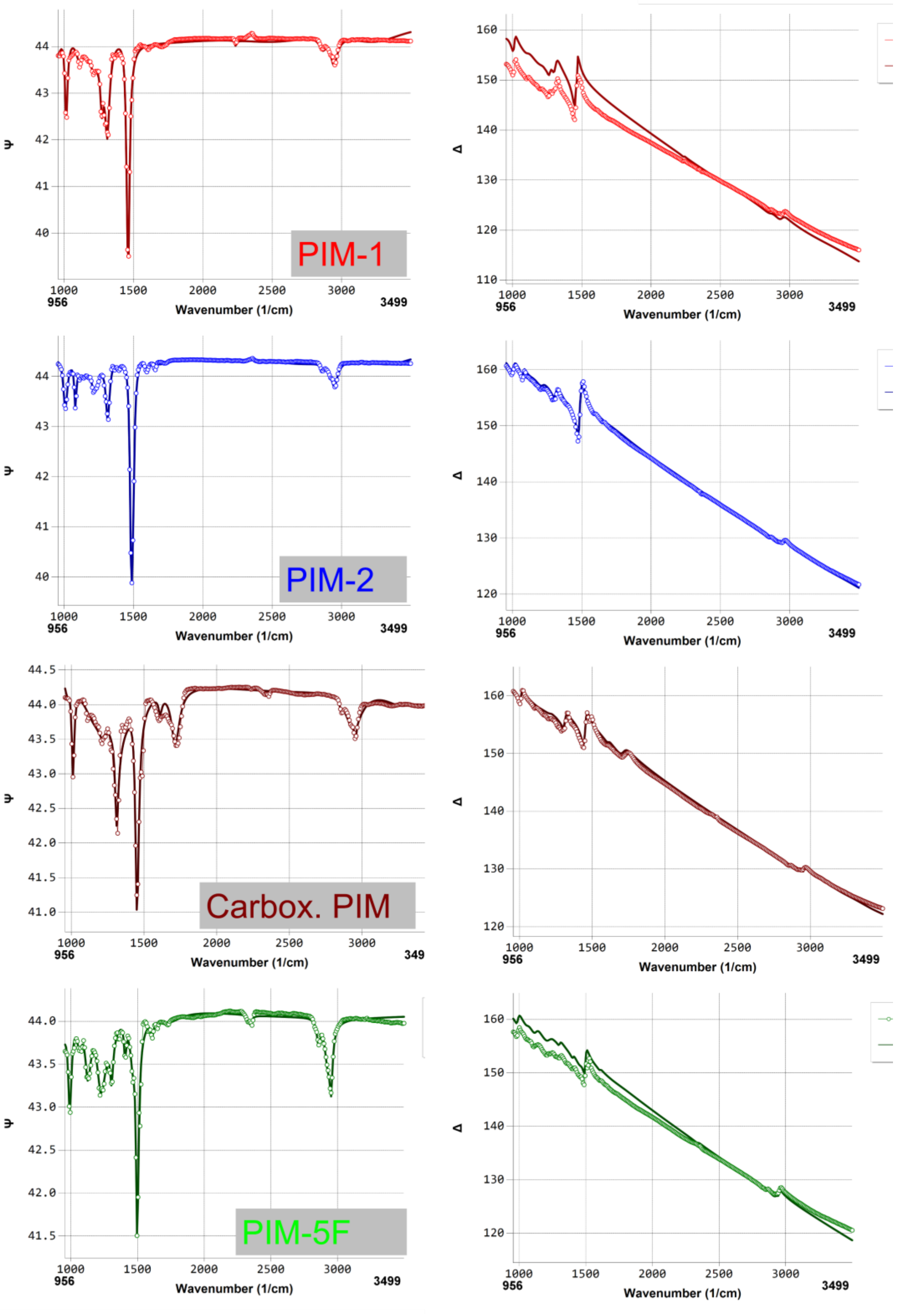

Figure S2. Measured (empty symbols) and fit generated (solid line) FT-IR ellipsometric spectra of PIM samples spin-coated on Au.

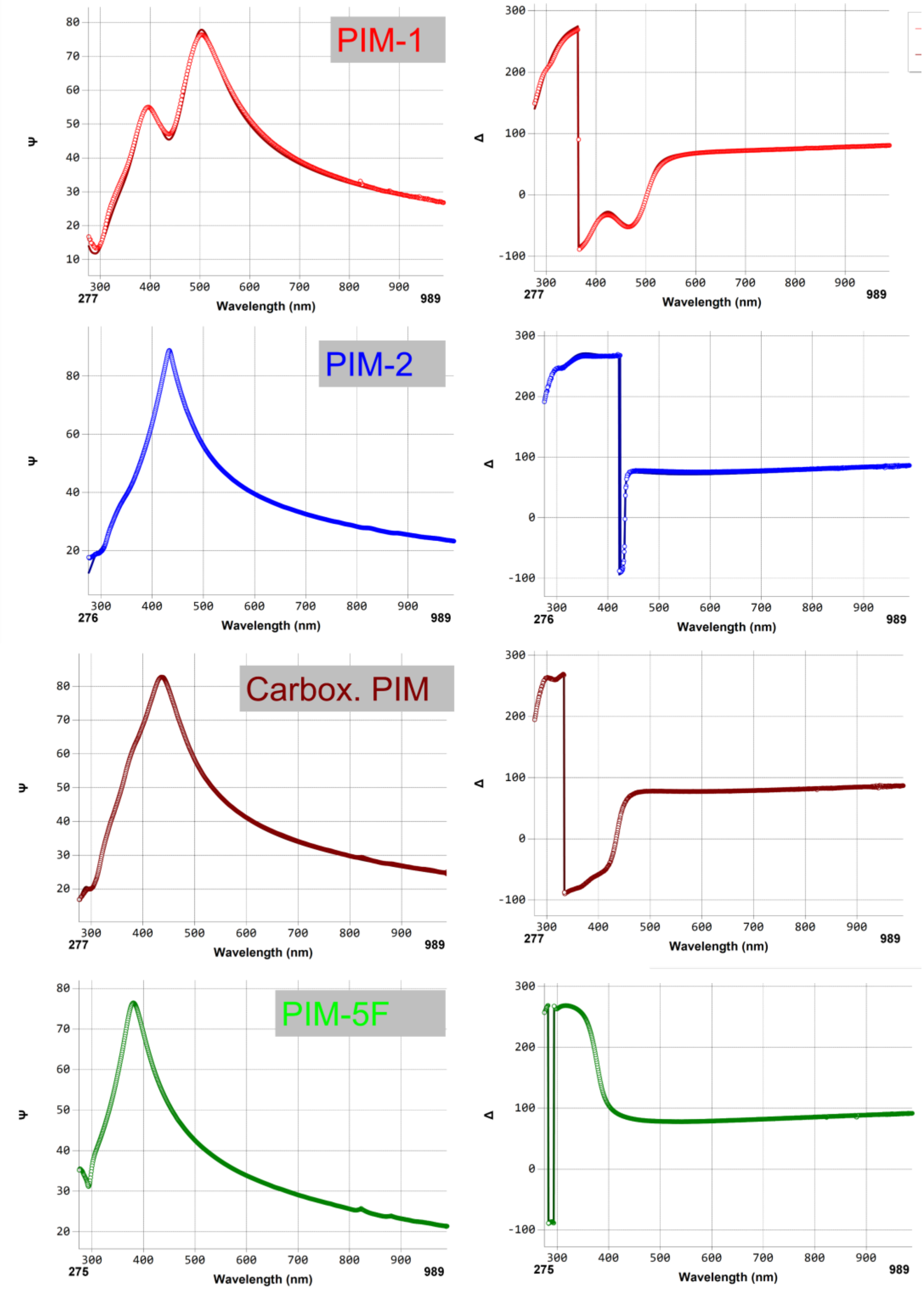

Figure S3. Measured (empty symbols) and fit generated (solid line) UV-Vis ellipsometric spectra (at P/P^0=0) of PIM samples spin-coated on Si.

Individual *n* and *h* isotherms (from EP)

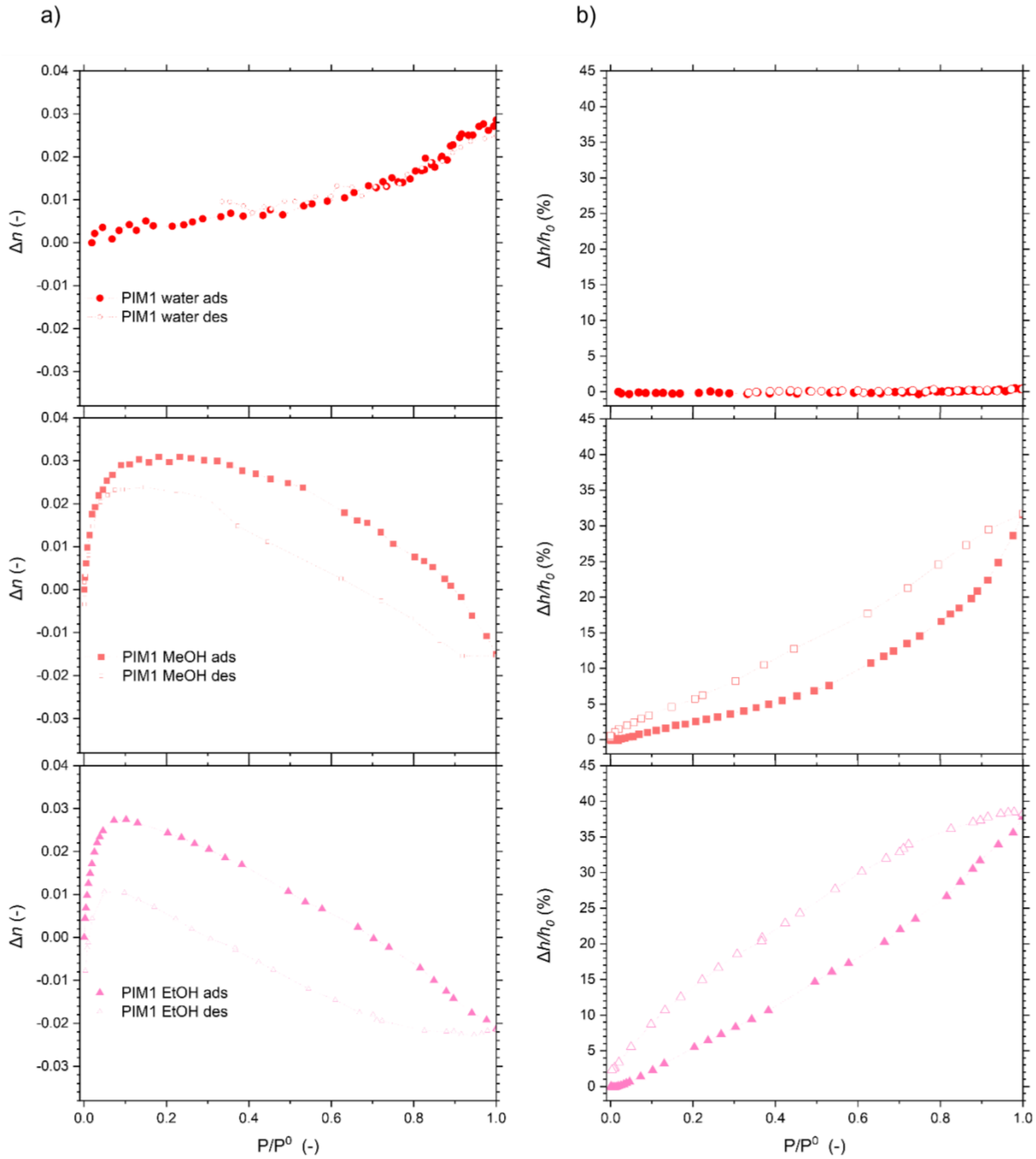

Figure S4. PIM-1 n isotherms (a) and h isotherms (b) for water, methanol and ethanol (full cycle, n taken at λ=632 nm).

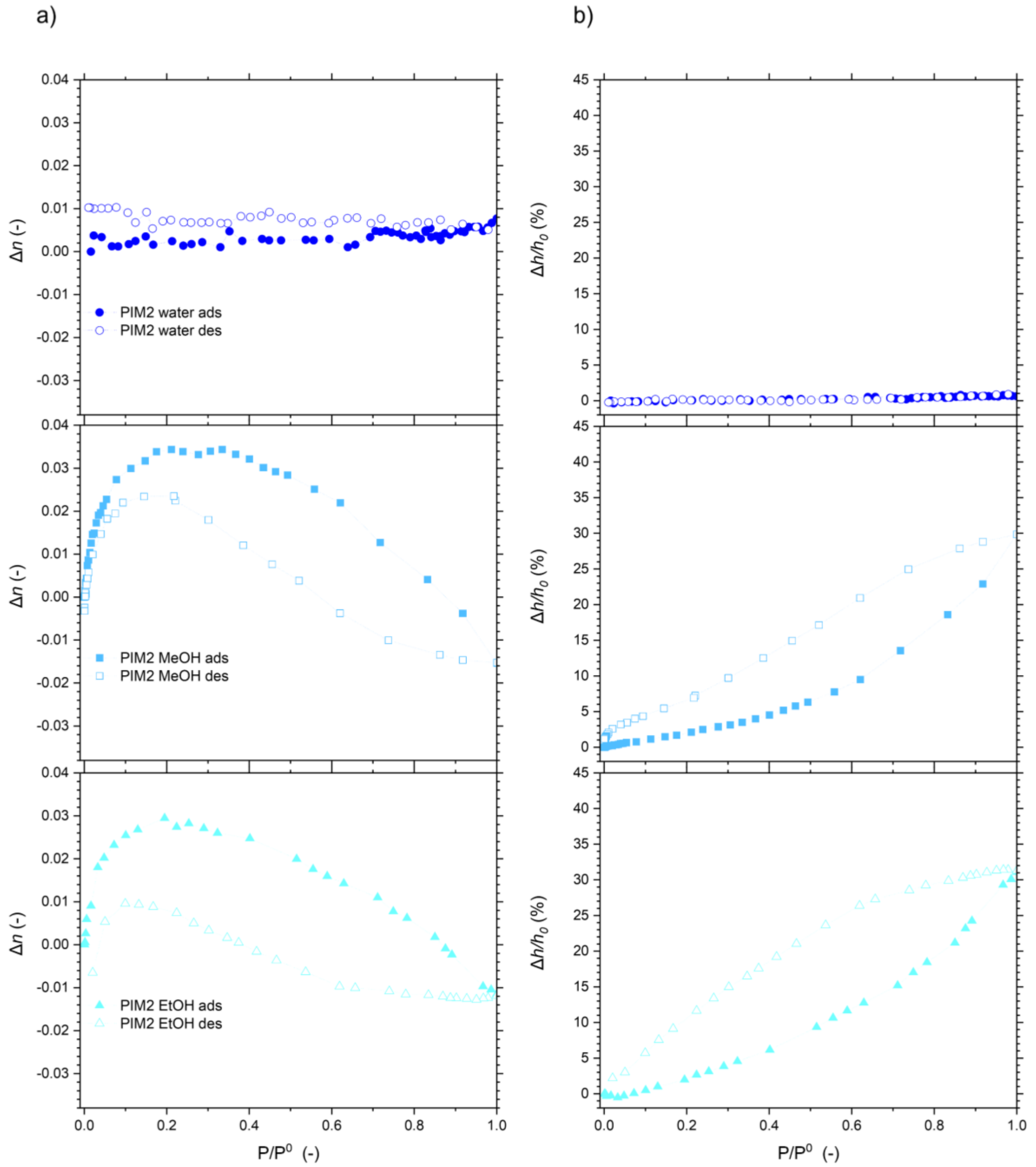

Figure S5. PIM-2 *n* isotherms (a) and *h* isotherms (b) for water, methanol and ethanol (full cycle, *n* taken at λ=632 nm).

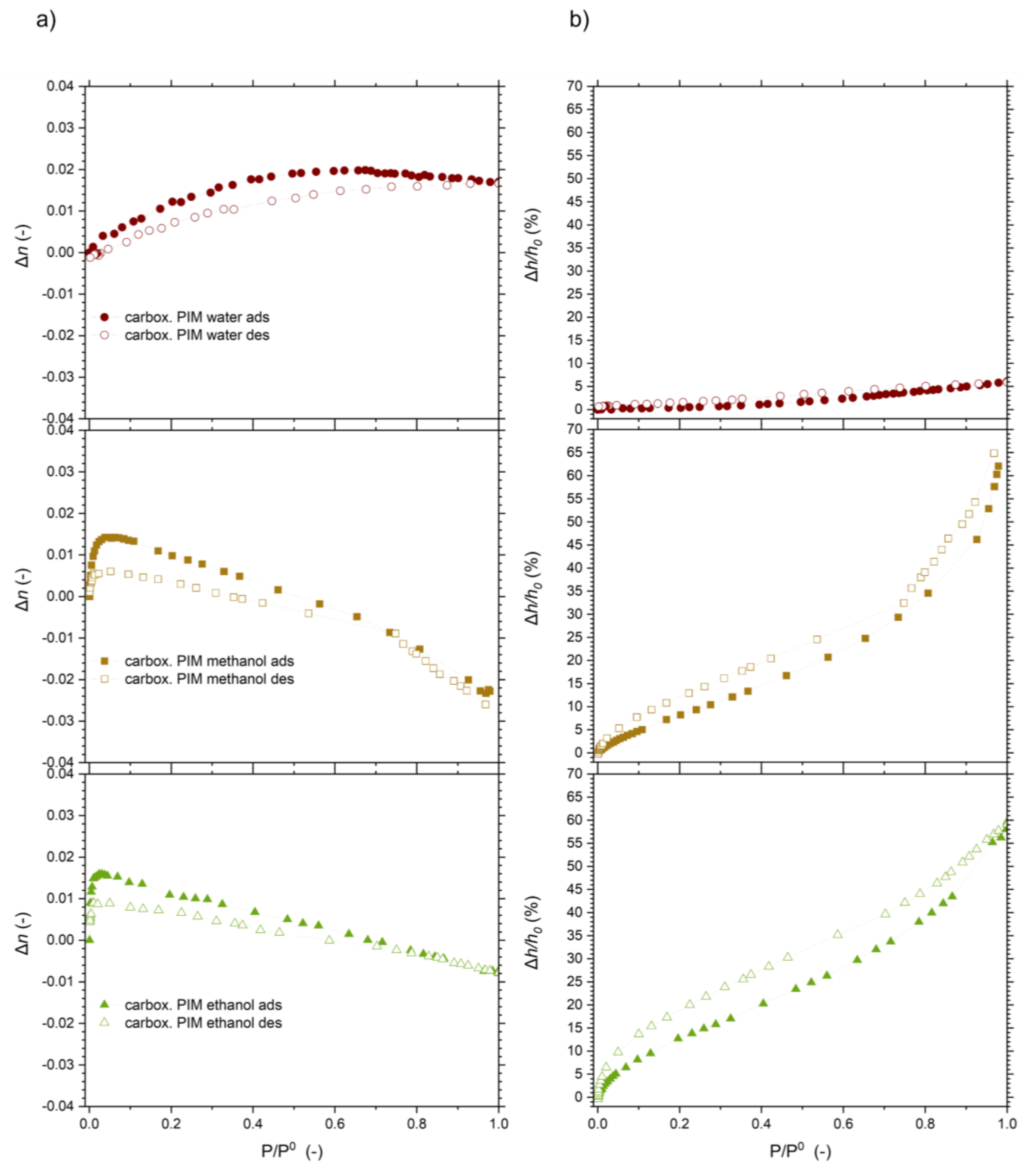

Figure S6. cPIM-1 n isotherms (a) and h isotherms (b) for water, methanol and ethanol (full cycle, n taken at λ=632 nm).

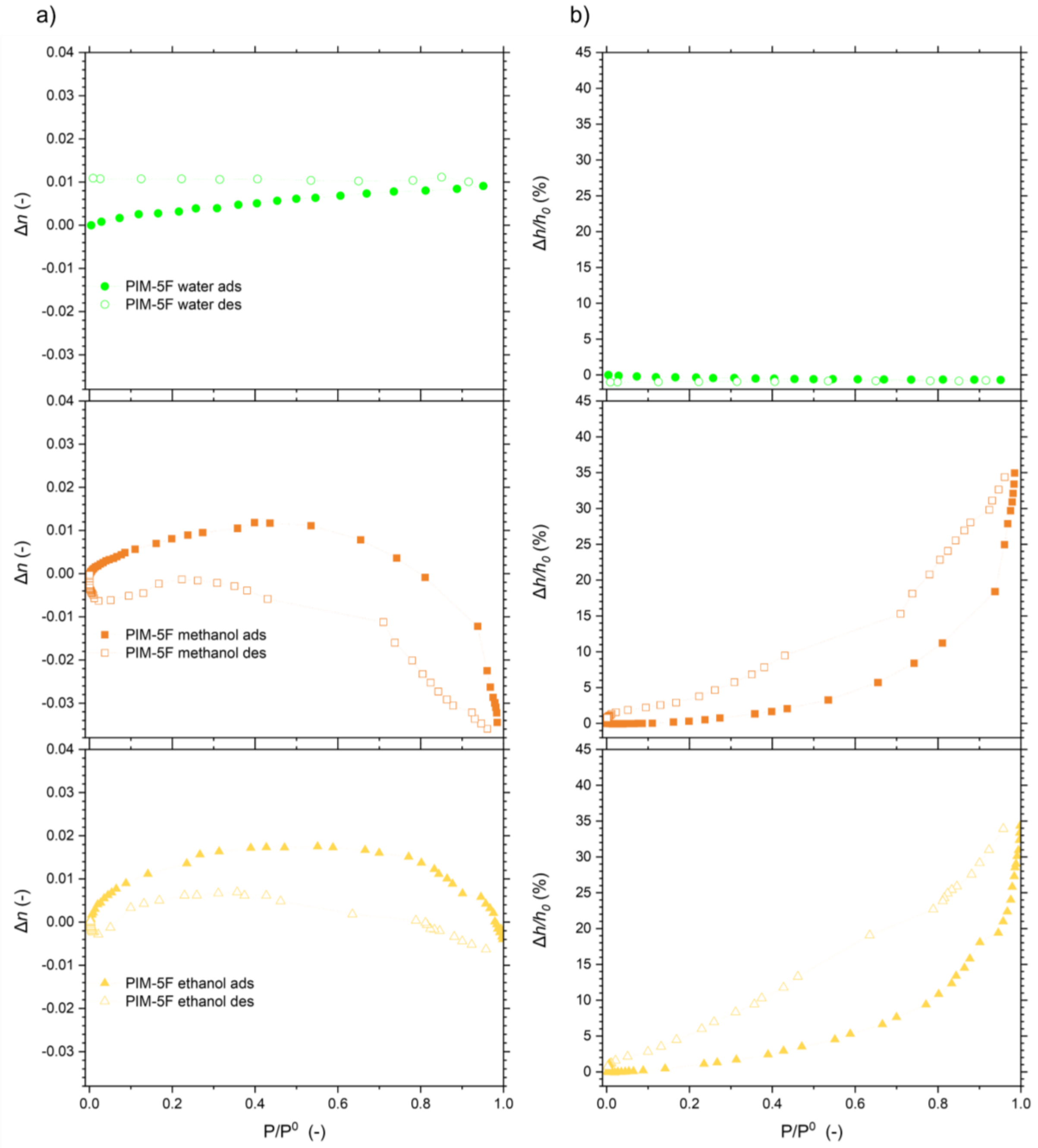

Figure S7. PIM-5F *n* isotherms (a) and *h* isotherms (b) for water, methanol and ethanol (full cycle, *n* taken at λ=632 nm).

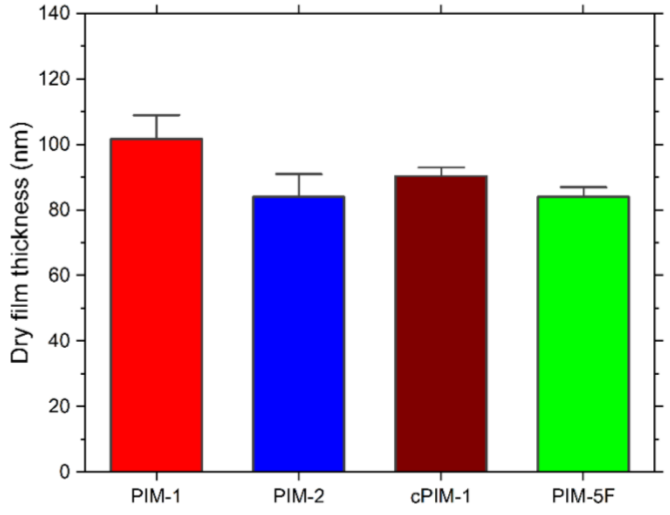

Figure S8. Thickness statistics for spin-coated polymers (with sample size = 3 for each different PIM). Samples were measured immediately after spin-coating in vacuum chamber with ellipsometry.

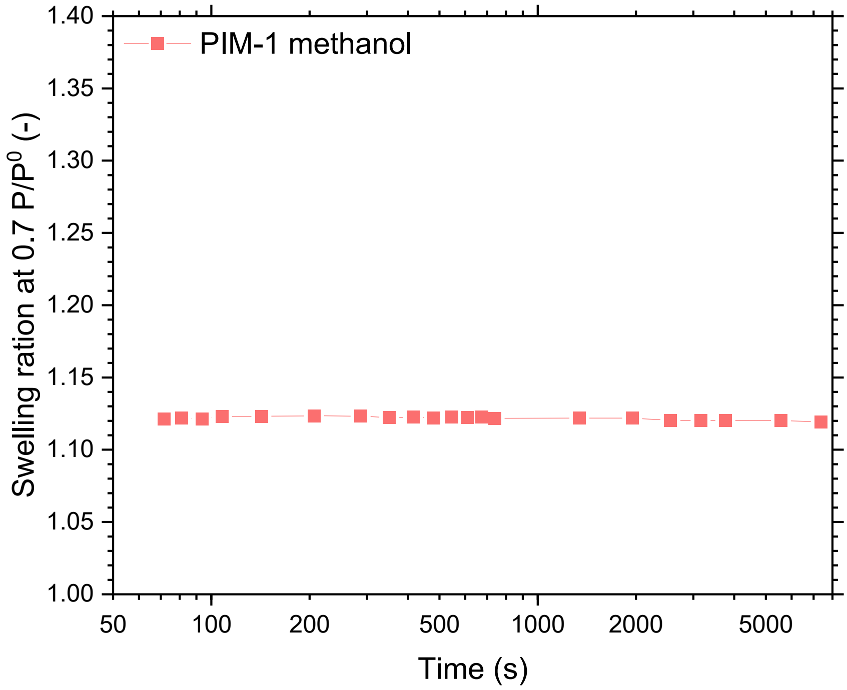

Figure S9. Thickness change ratio of PIM-1 in methanol vapor in time.

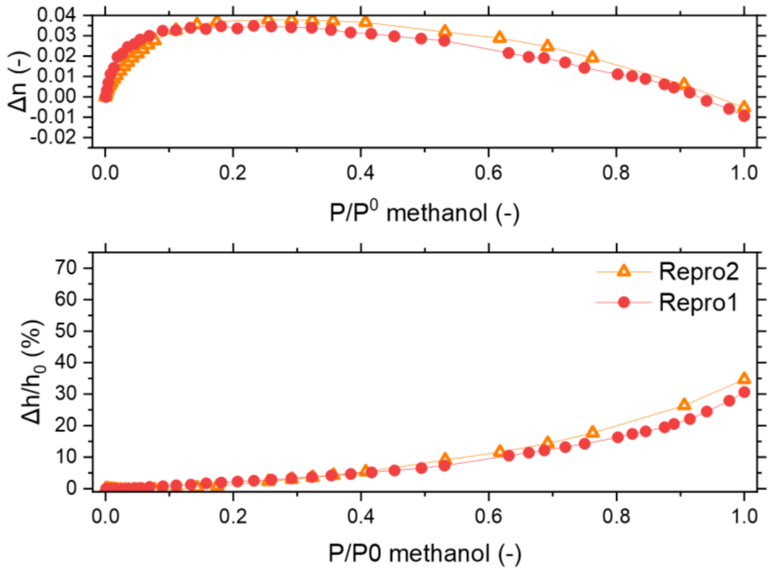

Figure S10. Reproducibility testing of EP cycles. Two separate PIM-1 samples were measured immediately after spin-coating with methanol adsorptive. The two films were deposited and characterized two months apart from each other. The n and h isotherms are presented in the top and bottom respectively.

Table S1. Reproducibility of EP-derived structural characteristics on different samples

Test	**Thickes**	*n*	**Adsorption (MeOH**	**Absorption (MeOH**
1	95 nm	1.56	15%	32%
2	99 nm	1.54	14%	35%

Parameter sensitivity tests in effective medium fitting

In the Bruggeman effective medium approximation (EMA, equation 1 in main text), the sensitivity to matrix concentration becomes significantly more complex when the refractive index (represented by ε_{inf} in the optical model) of the matrix is also treated as a fitted parameter. This is because the Bruggeman EMA is a nonlinear mixing model where the effective refractive index n_{eff} of the composite is calculated based on the refractive indices and volume fractions of the individual components—i.e. the matrix, void and adsorptive phase.

When both the matrix refractive index $n_{polymer}$ and the void volume fraction are fitted simultaneously, strong parameter correlation arises. This means that small changes in one parameter can be compensated for by opposite changes in the other without significantly altering the modelled n_{eff}, and the goodness of fit, R^2. As a result, the fitting process becomes underdetermined or poorly conditioned in regions where the sensitivity of the model to one parameter cannot be decoupled from the other. This is demonstrated in **Figure S11.**, which showcases such a fit for PIM1-void-ethanol mixture.

In our case, parameter sensitivity is particularly problematic when the matrix is subject to swelling (which is due to vapor absorption, as described in **Figure 4**., main text), because the matrix refractive index itself can change with swelling. However, without an independent constraint (such as fixed $n_{polymer}$), the ellipsometric fit is not able to distinguish whether a decrease in n_{eff} is due to increased void fraction or due to a reduction in $n_{polymer}$. Consequently, the sensitivity of the fit to matrix concentration is reduced, and the resulting parameters becomes non-physical and highly uncertain. Thus, EMA fitting cannot describe these non-monotonous n_{eff} changes during the porosimetry measurement

To minimize this issue, the approach used in our work (see **Figure 5.**, main text) is to fix $n_{polymer}$ based on independent measurements (dry state refractive index) and fit only the ethanol+void volume fractions. Crucially, the fit is constrained to low relative pressure regions of the isotherm -where swelling is negligible- and the adsorption properties can be determined and subsequently extrapolated to the full pressure range.

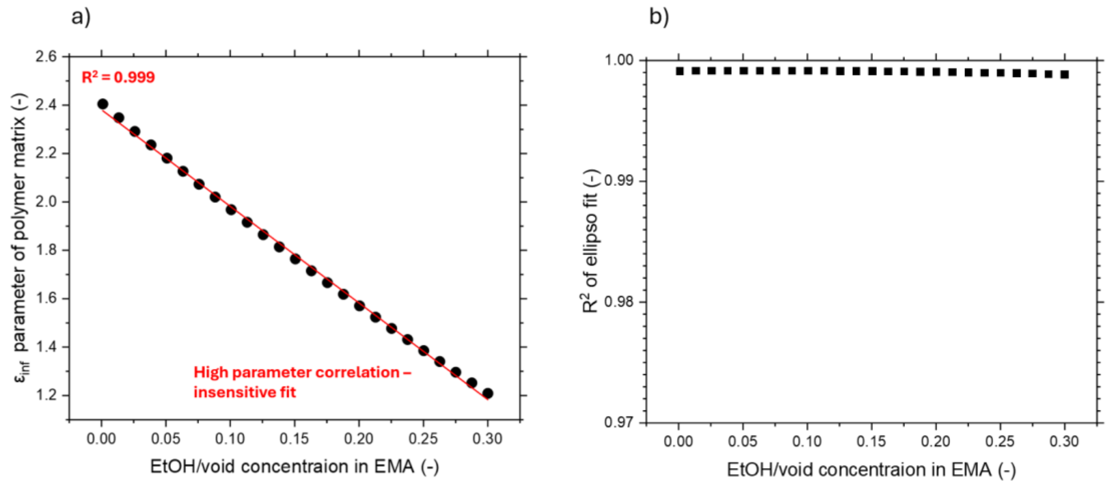

Figure S11. Parameter sensitivity analysis of the Bruggeman EMA with fitted polymer refractive index (a). Goodness of fit (R^2) results corresponding to these spectral fits (b).

Dual adsorption/absorption model

Consider a rectangular PIM film deposited on a substrate (see Fig. S9) and immersed in the adsorbate vapor at fixed temperature T and chemical potential μ. We assume that physical adsorption takes place in the micropores uniformly distributed throughout the entire volume. In addition, the gas molecules can be directly absorbed by the polymer matrix. Both adsorption and absorption are accompanied by the deformation process — the former leads to adsorption-induced deformation (AID) and the latter leads to polymer swelling. AID is modelled as a change in pore volume and is assumed to be independent of the polymer swelling. Additionally, we assume that the film can only vary in height as a result of deposition on the substrate. Thus, only approximately one-third of all pores can vary their dimensions.

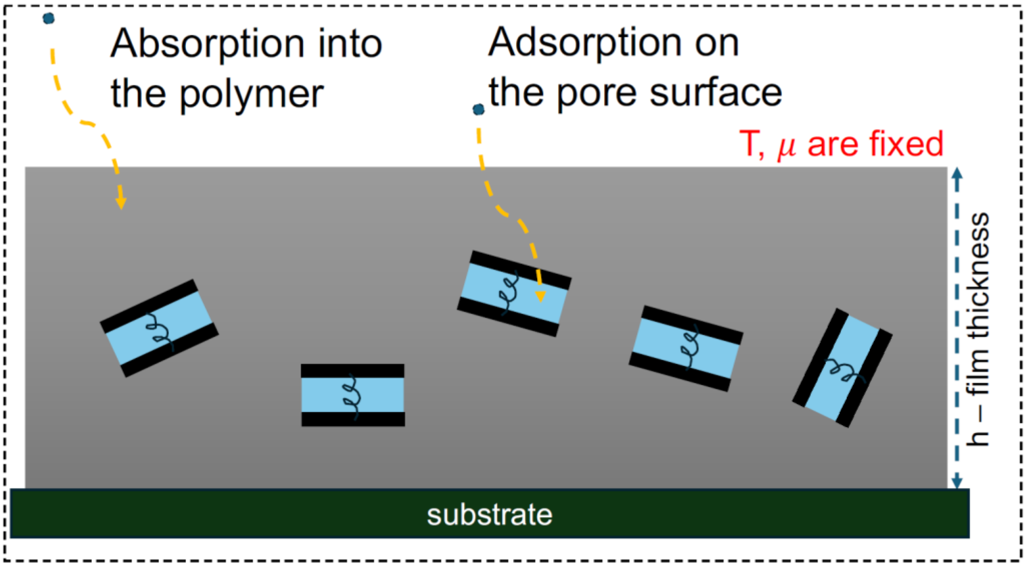

Figure S12. Schematic representation of the system under consideration. A PIM sample is assumed to have a rectangular shape, with film thickness h. The adsorbate molecules from the gas phase can be adsorbed on the pore surface and also be absorbed by the polymer matrix. We assume that the adsorption process takes place at constant T and μ.

The osmotic potential for the total system is written as:

$$\Omega_{\mathrm{s}} = F_{\mathrm{el}} + F_{\mathrm{ads}} + F_{\mathrm{p}}^{0} + F_{\mathrm{s}}^{0} + \Delta F_{\mathrm{mix}} - \mu N_{1}^{\mathrm{tot}} + pV \qquad (S1)$$

where the elastic contribution takes into account the pore deformation. We assume that each pore has a virtual spring, thus, the Helmholtz free energy can be approximated as: [1, 2]

$$F_{\mathrm{el}} = F_{\mathrm{el}}^{0} + \frac{N_{\mathrm{pores}}}{6} K \frac{(\Delta h_{\mathrm{p}})^{2}}{h_{\mathrm{p}}^{0}}, \qquad (S2)$$

here K is the effective elastic constant defining the pore response; h_{p} is the pore width; h_{p}^{0} is the pore width in the reference state. The adsorption process is described via a lattice gas approximation: [3]

$$F_{\mathrm{ads}} = -k_{\mathrm{B}}T[M \ln M - N \ln N - (M - N) \ln(M - N)] + \psi N, \quad (S3)$$

Where ψ is the interaction energy between the site on the polymer and the adsorbate molecule. F_{p}^{0} and F_{s}^{0} are the reference Helmholtz free energies of the pure polymer and liquid adsorbate, respectively. The mixing Helmholtz free energy is written in the Flory-Huggins approximation: [4, 5]

$$\Delta F_{\mathrm{mix}} = M_{\mathrm{ps}} k_{\mathrm{B}} T \left[\phi_{\mathrm{s}} \ln \phi_{\mathrm{s}} + \phi_{\mathrm{p}}/N_{\mathrm{p}} \ln \phi_{\mathrm{p}} + \chi \phi_{\mathrm{p}} \phi_{\mathrm{s}}\right], \quad (S4)$$

with χ being the Flory-Huggins interaction parameter.

The chemical potential of the adsorbate molecules in the gas phase is defined as:

$$\mu = \mu_0 + k_{\mathrm{B}} T \ln p / p_0, \quad (S5)$$

with μ_0 being reference chemical potential; k_{B} is the Boltzmann constant; p_0 is the saturation pressure at the fixed temperature. The number of polymers chains is constant, so we don't need to specify F_{p}^{0} dependencies. On the other hand, F_{s}^{0} is chosen to have the same chemical potential as the reference one for the adsorbate phase, μ_0. Thus,

$$F_{\mathrm{s}}^{0} = \mu_0 n_{\mathrm{s}} - p_0 n_{\mathrm{s}} v_0 \quad (S6)$$

with

$$V = \left(n_{\mathrm{s}} + n_{\mathrm{p}} N_{\mathrm{p}}\right) v_0 + V_{\mathrm{p}}. \quad (S7)$$

n_{p} is the number of polymer chains and N_{p} is the polymerization index.

$$\phi_{\mathrm{s}} = \frac{n_{\mathrm{s}}}{n_{\mathrm{s}} + n_{\mathrm{p}} N_{\mathrm{p}}} \quad (S8)$$

$$\phi_{\mathrm{p}} = \frac{n_{\mathrm{p}} N_{\mathrm{p}}}{n_{\mathrm{s}} + n_{\mathrm{p}} N_{\mathrm{p}}} \quad (S9)$$

We assume that adsorption and absorption take place independently, so we can split the osmotic potential into two parts and treat them separately. The adsorption osmotic potential as a function of two order parameters (N, h_{p}) can be written as:

$$\Omega_{\mathrm{ads}} = F_{\mathrm{el}} + F_{\mathrm{ads}} - \mu N + p V_{\mathrm{p}} \quad (S10)$$

Total pore volume is $V_{\mathrm{p}} = N_{\mathrm{pores}} v_{\mathrm{p}}$, where v_{p} is the volume of one pore. The maximum adsorbed amount is $M = N_{\mathrm{pores}}\left(N_{\mathrm{sites}}^{0} + \lambda \Delta h_{\mathrm{p}}/3\right)$, $\psi = \psi_0 + \kappa \Delta v_{\mathrm{p}}/3$. Minimizing Eq. S10 with respect to the order parameters we get two equations defining adsorbed amount and adsorption-induced deformation:[1]

$$\frac{\partial \Omega_{\mathrm{ads}}}{\partial N} = 0 \tag{S11}$$

$$\frac{\partial \Omega_{\mathrm{ads}}}{\partial h_p} = 0 \tag{S12}$$

Using Eqs. S11 and S12, after some algebra, we obtain the final equations:

$$\theta = \frac{bp/p_0}{1 + bp/p_0} \tag{S13}$$

$$\frac{\Delta h_{\mathrm{p}}}{h_{\mathrm{p}}^0} = -\tilde{\lambda} \ln(1 - \theta) - \tilde{\kappa}\theta \tag{S14}$$

where $b = e^{(\mu_0 - \psi)/k_B T}$, $\tilde{\lambda} = k_B T\lambda/K$, $\tilde{\kappa} = \kappa N_{\mathrm{sites}}/K$, N_{sites} is the number of accessible sites in one pore.

Now, minimizing the absorption osmotic potential

$$\Omega_{\mathrm{abs}} = F_{\mathrm{p}}^0 + F_{\mathrm{s}}^0 + \Delta F_{\mathrm{mix}} - \mu n_{\mathrm{s}} + p\left(n_{\mathrm{s}} + n_{\mathrm{p}} N_{\mathrm{p}}\right) v_0 \tag{S15}$$

with respect to the absorbed amount (n_{s}) we obtained the following equation on the absorbed fraction:

$$\ln(\phi_{\mathrm{s}}) + 1 - \phi_{\mathrm{s}} + \chi(1 - \phi_{\mathrm{s}})^2 = \ln(p/p_0)\,. \tag{S16}$$

The relative change of the polymer film, and thus the change in polymer volume can be defined as:

$$\frac{\Delta h}{h_0} = \frac{\Delta V}{V_0} = \left[\frac{\Delta h_{\mathrm{p}}}{h_{\mathrm{p}}^0}\frac{\varphi^0}{3} + \frac{\phi_{\mathrm{s}}}{1 - \phi_{\mathrm{s}}}(1 - \varphi^0)\right], \tag{S17}$$

here φ^0 is the porosity of the dry PIM.

Summarizing the above, we use Eqs. S13 and S16 to calculate the amount of adsorbed and absorbed. In its turn, Eq. S17 is used to estimate a relative change in the thickness of the PIM material. By means of five adjustable parameters ($\varphi^0, b, \tilde{\kappa}, \tilde{\lambda}, \chi$) we fitted the strain isotherms and the low pressure regions of the adsorption isotherms. The latter is important because of the limitations of effective medium models assuming no deformation during the sorption process. Thus, focusing on low pressures, we eliminate the most pronounced effect arising from absorption. In addition, the parameters used in our calculations are summarized in Tables S2, S3, S4.

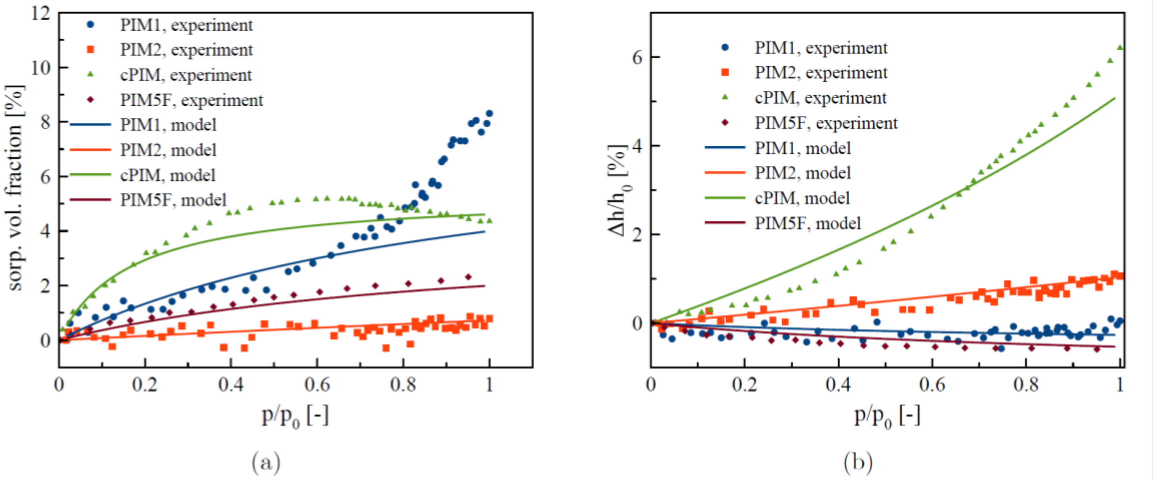

Figure S13. (a) Comparison of experimental and theoretical water volume fraction in the PIMs. (b) Comparison of experimental and theoretical strains during water sorption in the PIMs. The model parameters are given in Table S1.

Table S2. Parameters corresponding to H_2O data.

Material	$\boldsymbol{\varphi^0}$	$\boldsymbol{b}$	$\tilde{\kappa}$	$\tilde{\lambda}$	χ
PIM-1	0.08	1	0.2	0	-
PIM-2	0.08	0.1	0	0	3.588
*c*PIM-1	0.0538	6	0	0	2.24
PIM-5F	0.04	1	0.8	0	-

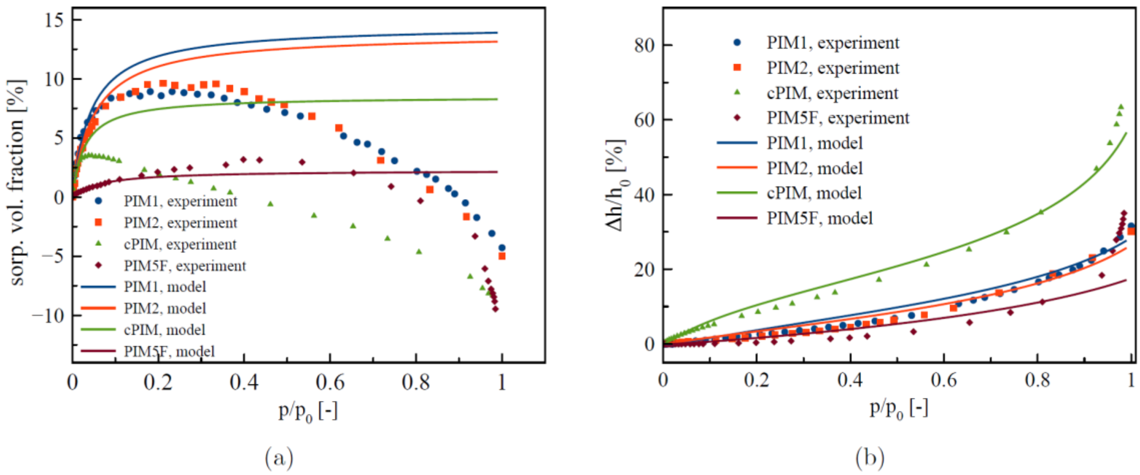

Figure S14. (a) Comparison of experimental and theoretical methanol volume fraction in the PIMs. (b) Comparison of experimental and theoretical strains during methanol sorption in the PIMs. The model parameters are given in Table S2.

Table S3. Parameters corresponding to MeOH data.

Material	$\boldsymbol{\varphi^0}$	$\boldsymbol{b}$	$\tilde{\kappa}$	$\tilde{\lambda}$	χ
PIM-1	0.145	23	0.85	0.6	1.225
PIM-2	0.138	20	0.25	0.3	1.225
*c*PIM-1	0.085	35	1.7	2.0	0.99
PIM-5F	0.0226	15	1.3	0.45	1.44

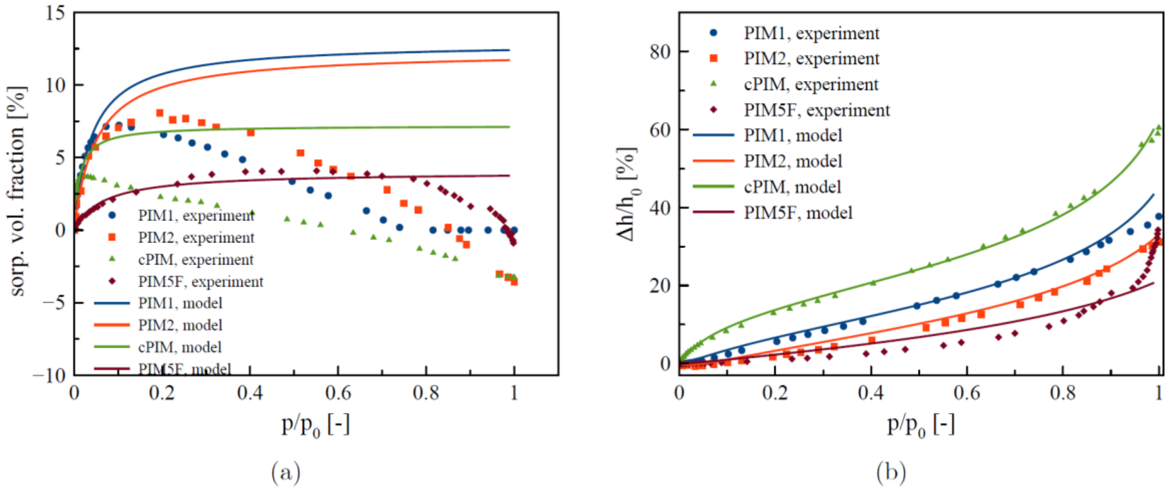

Figure S15. (a) Comparison of experimental and theoretical ethanol volume fraction in the PIMs. (b) Comparison of experimental and theoretical strains during ethanol sorption in the PIMs. The model parameters are given in Table S3.

Table S4. Parameters corresponding to EtOH data.

Material	$\boldsymbol{\varphi^0}$	$\boldsymbol{b}$	$\tilde{\kappa}$	$\tilde{\lambda}$	χ
PIM-1	0.129	25	1.0	1.0	1.05
PIM-2	0.123	20	1.5	0.9	1.155
*c*PIM-1	0.072	80	1.5	2.1	0.99
PIM-5F	0.04	15	1.0	0.7	1.36

Pore size estimation from D-R model

Mean micropore width (w) is calculated using the empirical relation between heat of sorption (E) and pore size

$$w = \frac{K_{Benzene}}{E_{Benzene}}, \qquad (S18)$$

where $K_{Benzene}$ is determined to be 12000 $nm\frac{J}{mol}$ for this reference adsorptive.[6] Generally,

for adsorbtives other than benzene, the isosteric heat of sorption can be expressed as βE_0. Herein β is an affinity parameter normalized to the specific reference adsorptive (i.e. $\beta_{\text{benzene}} = 1$). The values of β can be approximated based on the ratio of parachors or the ratio of molar masses of the specific adsorptives. Thus, pore size can be estimated by any adsorptive without the need to determine *K*, as follows:

$$w = \frac{\beta K_{Benzene}}{\beta E_0} = \frac{B_{micro} * RT}{\beta E_0} = B_{micro}\sqrt{\left(\frac{RT}{\beta E_0}\right)^2}, \qquad (S19)$$

where B_{micro} is introduced as a microporous coefficient of the adsorptive, with the definition of:

$$B_{micro} = \frac{\beta K_{Benzene}}{RT}. \qquad (S20)$$

Using equation S19, micropore size can be estimated in a straightforward way, since $\left(\frac{RT}{\beta E_0}\right)^2$ is directly acquired from the slope of the fitted logarithmic D-R equation to the experimental data (equation 5 in main text). Moreover, the microporous coefficient parameters only depend on temperature, and β, which can be approximated, or determined experimentally[7] (i.e., typical values include $B_{micro,water} = 0.80$, $B_{micro,methanol} = 1.80$, $B_{micro,ethanol} = 2.59$).

The DR model can be expanded to obtain a pore size distribution (PSD) as a function of x (varying pore size in nm), which is represented by gaussian curves.

$$PSD = \frac{V_t}{V_{max}}\exp\left\{-\frac{\ln^2\left(\frac{x}{w}\right)}{\ln\left(\frac{w}{2r_{mol}}\right)}\right\} \qquad (S21)$$

Where$V_{max} = V|_{\frac{P}{P0}=1}$and r_{mol} is the adsorptive molecular radius ($r_{mol,methanol} = 0.18$ nm, $r_{mol,ethanol} = 0.22$ nm).

References

(1) Kolesnikov, A.; Georgi, N.; Budkov, Y. A.; Möllmer, J.; Hofmann, J.; Adolphs, J.; Gläser, R. Effects of enhanced flexibility and pore size distribution on adsorption-induced deformation of mesoporous materials. *Langmuir* **2018**, 34, 7575–7584.

(2) Ustinov, E.; Do, D. Effect of adsorption deformation on thermodynamic characteristics of a fluid in slit pores at sub-critical conditions. *Carbon* **2006**, 44, 2652–2663.

(3) Hughes, A. P.; Thiele, U.; Archer, A. J. An introduction to inhomogeneous liquids, density functional theory, and the wetting transition. *American Journal of Physics* **2014**, 82, 1119–1129.

(4) Knychala, P.; Timachova, K.; Banaszak, M.; Balsara, N. P. 50th anniversary perspective: phase behavior of polymer solutions and blends. *Macromolecules* **2017**, 50, 3051–3065.

(5) Favre, E.; Trong Nguyen, Q.; Schaetzel, P.; Clément, R.; Néel, J. Sorption of organic solvents into dense silicone membranes. Part 1. —Validity and limitations of Flory–Huggins and related theories. *Journal of the Chemical Society, Faraday Transactions* **1993**, 89, 4339–4346.

(6) Dubinin, M.M. Generalization of the theory of volume filling of micropores to nonhomogeneous microporous structures. *Carbon N. Y.* **1985**, 23, 373–380.

(7) Wood, G.O. Affinity coefficients of the Polanyi/Dubinin adsorption isotherm equations: A review with compilations and correlations. *Carbon* **2001**, 39, 343-356
